# Extra Territorial Excursions by European badgers are not limited by age, sex or season

**DOI:** 10.1038/s41598-020-66809-w

**Published:** 2020-06-15

**Authors:** David J. Kelly, Aoibheann Gaughran, Enda Mullen, Teresa MacWhite, Peter Maher, Margaret Good, Nicola M. Marples

**Affiliations:** 1 Department of Zoology, School of Natural Sciences, Trinity College Dublin, DO2 CX56 Dublin, Ireland; 2 National Parks and Wildlife Service, Department of Culture, Heritage and the Gaeltacht, 90 North King Street, Smithfield, Dublin, D07 N7CV Ireland; 3 Department of Agriculture, Food and the Marine, Kildare Street, Dublin, DO2 WK12 Ireland

**Keywords:** Community ecology, Ecological networks, Behavioural ecology

## Abstract

European badgers (Meles meles) in medium and high density populations show strong territorial behaviour. Territories in these populations are contiguous, well-marked and often unchanging over many years. However, badgers do not always stay within their territorial boundaries. In our medium-density population, most individual badgers made extra-territorial excursions (ETEs) throughout the year. ETEs were most frequent between April and September and least frequent in December and January (the period of winter lethargy). Male badgers made longer and more frequent ETEs than females (especially between January and March, and in autumn). Breeding females made longer and more frequent ETEs than non-breeding females in November. While these peaks correspond with the main mating seasons, mating activity does not explain ETEs throughout the year. The shorter, but more frequent, ETEs in summer months may serve a monitoring purpose, rather than simply providing additional mating opportunities with badgers from outside the ‘home’ social group. We found that young badgers did not make regular ETEs until the summer of their second year. If badgers could be vaccinated as cubs, this would reduce any potential risk of TB spread during ETEs.

## Introduction

In the classic view of badger social organisation^1,2^, badgers live in social groups, which share a communal territorial space. Boundaries between adjoining social groups are marked by latrines. These latrines are often linked by well-trodden paths, which suggest that badgers patrol the borders between territories^1^. If such territorial behaviour were strictly maintained, then, with the typically low dispersal rates reported in badgers (Table 1), this should lead to major inbreeding. However, relatedness within social groups is much lower than this strict territorial behaviour predicts^3^.

**Table 1:** Dispersing badgers as a proportion of population – Irish and UK studies.

proportion	activity reported	area of study	authors
22.1%	permanent movers	Woodchester Park, UK	Rogers *et al*.^4^
20.0%	dispersed	Wytham Woods, UK	Pope *et al*.^3^
34.0%	intergroup movements	Brighton, UK	Huck *et al*.^5^
18.0%	dispersal	Wicklow, Ireland	Gaughran *et al*.^6^

Observational evidence has identified badgers of both sexes making temporary visits to other social groups for mating purposes^7,8^. These visits have been described as extra-territorial excursions (ETEs)^8^, as the badger leaves their home territory and enters the territory of a different social group.

Research conducted in Wytham Woods, Oxfordshire (a very high density population) showed that just less than 50% of cubs in any given social group were sired by males from within that group^9^. Furthermore, a single litter could contain cubs sired by males from different social groups^10^. This identified a polygynandrous mating system in badgers, i.e. both males and females have multiple mating partners during a breeding season. As female badgers can delay the implantation of fertilised eggs^8^ and allow the development of different embryos at different times (i.e. superfetation)^11^, they are released from the limitation of seasonal breeding cycles. It would, therefore, appear that female badgers would be as likely as males to benefit from visits to other social groups for extra-group matings, at any time of year.

Territorial, group-living species are susceptible to inbreeding depression^12^, which can reduce fitness^13^, productivity^14^, and longevity^15^. For these reasons, such species may seek to avoid inbreeding^16^, through kin recognition^17^, dispersal^10^, extra-group mating^9^, and reproductive suppression^18^.

ETEs allow opportunities for extra-group mating, as well as assessment of the resources and occupants of neighbouring territories, which may facilitate dispersal. ETEs (also known as forays or incursions) have been observed in European badgers^19^, Eurasian beavers (*Castor fiber*)^20^, American beavers (*Castor canadensis*)^21^, meerkats (*Suricata suricatta*)^22^, red foxes (*Vulpes vulpes*)^23^, Arctic foxes (*Vulpes lagopus*)^24^, Kit foxes (*Vulpes macrotis*)^25^, wolves (*Canis lupus*)^26^ and a number of primate species.

Work at Wytham Woods, following on from the genetic revelations of polygynandry^10^, identified nondispersal intergroup movements by badgers^27^. However, as these data relied on capturing badgers, rather than tracking them, it is not clear how many of those movements represent ETEs. More recently, a study of a high density population of badgers in Cornwall, UK^28^, showed that badgers make ETEs throughout the year, with peaks in February and September. A study at Woodchester Park (Gloucestershire) has also demonstrated seasonal variation in social interactions between social groups^29^. This indicates that classical territorial borders^2,30^ are frequently breached by ETEs, so are far more permeable than originally thought.

In Ireland, where densities are between 1 and 2 badgers per km^2^, badgers have been recorded making ETEs in all seasons^31,32^. In a large scale Irish trapping study^33^ it was noted that males ‘dispersed’ more frequently than females, but that females made proportionally more long-distance dispersal attempts than males. Byrne *et al*. ^33^found that movements of >1 km (identified as “extra-patch” movements) accounted for 57% of all movements detected. Unfortunately, it was not possible to distinguish between ETEs and dispersal-type movements in that study. By contrast, O’Mahony^32^ found that while inter-group interactions were occasionally recorded, they were too few (0.35% of contacts) for formal analysis. However, while the proximity collars in that study identified when two collared badgers were within 2 m of each other, the collars could not identify when badgers left their home territory (an ETE), but did not encounter another collared badger.

Using our current knowledge of ETEs, we can make some predictions:

1. ETEs are an obvious way of facilitating outbreeding in a species that behaves territorially, and so faces potential risks of inbreeding. We would therefore expect any seasonal peaks in ETEs to match peaks in breeding activity, if outbreeding were common.
2. If ETEs serve a purpose other than outbreeding, we would anticipate a background rate of ETEs outside the seasonal breeding peaks.
3. Delayed implantation and superfetation allow female badgers to mate throughout the year. As dispersal in badgers is relatively uncommon, we might expect female badgers (as well as males) to make ETEs in order to secure extra-group matings that allow outbreeding.
4. In addition, as males tend to disperse to territories adjacent to their natal group, we would expect older females to make longer ETEs than younger females, in order to ensure outbreeding.

Here we report, in far more detail than previous studies, the patterns of ETEs recorded from a medium density population of badgers. The study spanned six years, between 2010 and 2016 in County Wicklow, Ireland. Working with data from 83 badgers, we have been able to provide a thorough assessment of the distance and frequency of ETEs made by individuals of known age and sex throughout the year, and derive an ETE ‘investment’ metric, giving insight into the probable functions of ETEs in badgers.

## Results

**Figure 1:**
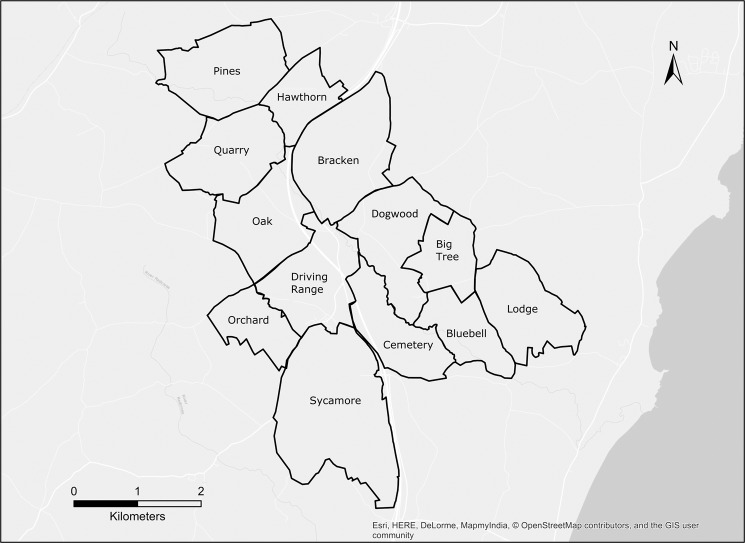
Map of the study area, illustrating the territorial boundaries of the focal social groups and the contiguous nature of their arrangement. The base image for this figure was produced by OpenStreetMap. Credit: OpenStreeMap contributors. This image is licensed under Open Database License, “ODbL” 1.0. The license terms can be found at the following link: https://wiki.osmfoundation.org/wiki/Licence. The figure was produced using ArcGIS 10.4.1 (Esri, Redlands, CA).

Using the tracking data from our badgers, we determined that social group territories were contiguous across the study area (Fig. 1). While there was some rearrangement of territorial borders from year to year, and also territory size (Table S1), the relative position of the social groups was constant across the study period. As social group territories were contiguous, any badgers leaving their own territory would automatically enter the territory of another social group.

The data from the study were considered in three ways: (1) the proportion of male and female badgers making ETEs across the year; (2) the frequency with which the badgers in the study made ETEs, and how that varied with season, age and sex; (3) the distance of ETEs made by badgers of each age and sex cohort, and how that varied with season.

### Proportion of badgers making ETEs

**Figure 2:**
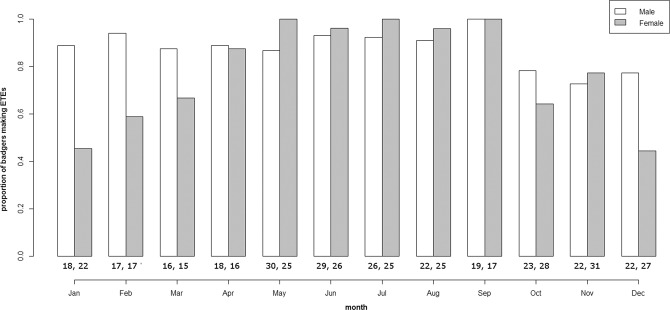
Proportion of male and female badgers making Extra-Territorial Excursions during each month. Sample sizes for cohorts are given below each bar.

We collected movement data from 83 individuals (44 males, 39 females) during the study period (April 2010 - August 2016). We found that most of our collared badgers made ETEs throughout the year (Fig. 2). Indeed, over half of the collared cohort made ETEs in any given month of the year, and all of our study badgers made ETEs in September. Male badgers showed a short period of relatively low ETE activity as they were fattening for winter lethargy (73–78% of males making ETEs between October and December, compared to 87–100% of males making ETEs across the rest of the year). In contrast, female badgers showed a longer period of lower ETE activity (44–67% of females made ETEs between October and March), which contrasted with a period of higher ETE activity (88–100% of females made ETEs between April and September). During the winter/spring period (October to March), there was a lower proportion of females making ETEs than males (Wilcoxon signed rank test: W = 34.5, p-value = 0.010). However, there was no difference between the proportion of male and female badgers making ETEs in the summer/autumn period (April to September) (Wilcoxon signed rank test: W = 8.5, p-value = 0.14).

### Frequency of ETEs

**Figure 3:**
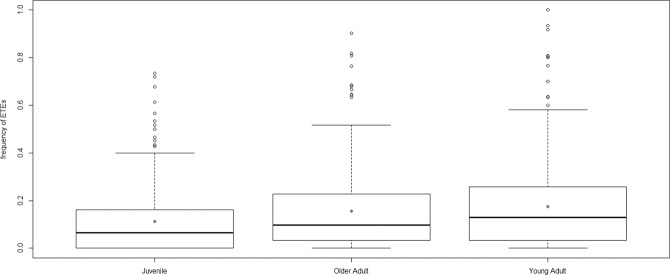
Frequency of extra-territorial excursions made by different age cohorts of badgers within the study period: juveniles (cubs and yearlings), young adults (2 and 3 year olds) and older adults (older than 3 years). Boxes represent the second and third quartiles, while bars within these boxes represent median values and black filled circles represent mean values. Whiskers extend up to 1.5 times the interquartile range (Q3 – Q1) from the boxes. Data points outside the range of the whiskers are represented individually by open circles. Dark grey dots mark the mean values for each age cohort.

The age of the badger and the month of the year were good predictors of the frequency of ETEs made by badgers (independent effects of **age.cohort** and **month** - Table 2). *Young Adult* badgers (2 and 3 year olds) made more frequent ETEs than *Juveniles* (cubs and yearlings) (post-hoc tests Table S2) or *Older Adult*s (>3 years old) (post-hoc tests Table S2) (Fig. 3). Within the *Juvenile* cohort, yearlings (median = 0.07, mean = 0.12) made more frequent ETEs than cubs (median = 0.00, mean = 0.06), but these differences were not statistically significant.

**Table 2:** ANOVA results from the best (lowest AICc) model (GLMM) explaining variation in ETE frequency (N = 838 monthly assessments).

	estimate	Std. Err.	Z	P > z	
age cohort	0.06776	0.01817	3.729	0.000192	***
month	0.17802	0.02245	7.929	2.21E-15	***
sex	0.05059	0.02588	1.955	0.050614	NS

**Figure 4:**
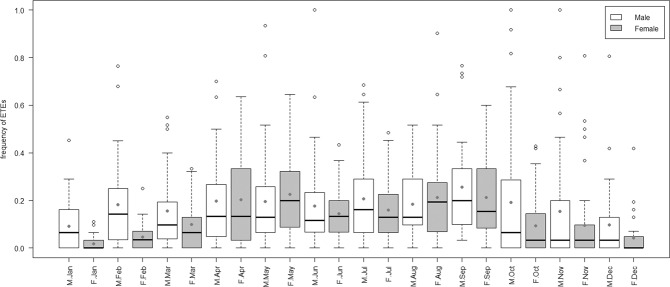
Frequency of extra-territorial excursions made by male (M.month) (empty boxes) and female (F.month) (pale grey boxes) badgers across the year. Boxes represent the second and third quartiles, while bars within these boxes represent median values. Whiskers extend up to 1.5 times the interquartile range (Q3 – Q1) from the boxes. Data points outside the range of the whiskers are represented individually by open circles. Dark grey dots mark the mean values for each sex/month cohort.

Badgers appeared to have two discrete periods of extra-territorial activity, as evidenced by ETE frequency (post-hoc tests – Table S3); a higher activity period, with more frequent ETEs, (April to September) and a lower activity period, with less frequent ETEs (October to March) (Fig. 4). While **sex** was included as an independent term in the best model (Table 1), the correlation between the sex of the badger and the frequency with which they made ETEs did not quite reach significance (P = 0.0506, Table 2) across the whole year, although the trend was towards males making, on average, more frequent ETEs throughout the year (Fig. 4 – mean values). While male and female badgers made ETEs with similar frequency between April and September, female badgers made fewer ETEs than male badgers between October and March (Fig. 4, post-hoc tests Table S3).

### ETE distance

Male badgers, on average, made longer ETEs than females (median ETE distances: Male = 131.5 m, Female = 87.1 m, mean ETE distances: Male = 290.0 m, Female = 178.0 m, Figure S1), although this difference was most apparent between October and April (**month**:**sex** interaction term – Table 3, post-hoc tests Table S4). In other words, males made longer ETEs than females, but this difference was less marked in the summer, when both sexes made relatively short ETEs (independent effect of **month** – Table 3). *Juvenile* badgers (mean = 321.6 m, median = 154.6 m) made longer journeys than *Young Adult*s (mean = 225.2 m, median = 109.2 m) or *Older Adult*s (mean = 215.0 m, median = 90.7 m) (post-hoc tests – Table S5). Within the *Juvenile* cohort, yearlings (mean = 337.8 m, median = 157.4 m) made longer journeys than cubs (mean = 173.6 m, median = 129.2 m) (Figure S2). Furthermore, cubs rarely made ETEs in excess of 500 m, while yearlings frequently did so (Figure S2).

**Table 3:** ANOVA of the best (lowest AIC) model (GLMM) explaining variation in log(ETE distance) (N = 3707 ETEs from 838 monthly assessments). month = calendar month; age cohorts used were Juvenile (age = 0, 1), Young Adult (age = 2, 3), Older Adult (age > 3).

	Chi Sq.	df	Pr(>Chisq)	
(Intercept)	194.5	1	<2.2E-16	***
age.cohort	4.8	2	0.09253	
month	48.5	11	1.17E-06	***
sex	4.2	1	0.04146	*
age.cohort: month	84.2	22	3.29E-09	***
month: sex	46.0	11	3.24E-06	***

**Figure 5:**
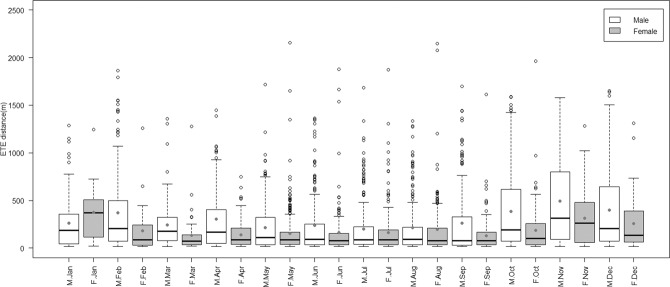
Distance of extra-territorial excursions made by male (M.month) (empty boxes) and female (F.month) (pale grey boxes) badgers across the year. Boxes represent the second and third quartiles of the data, while bars within these boxes represent median values. Whiskers extend up to 1.5 times the interquartile range (Q3 – Q1) from the boxes. Data points outside the range of the whiskers are represented individually by open circles. Dark grey dots mark the mean values for each sex/month cohort.

Female badgers made their longest ETEs in January (mean = 374 m, median=370, N = 15), while male badgers made their longest ETEs in November (mean = 494 m, median=314 m, N = 173) (Fig. 5). However, female badgers also made long ETEs in November (mean = 313 m, median = 263 m, N = 128).

Considering ETE distance and frequency together, badgers appear to make less frequent (monthly median range: 0.016–0.10, Table S6), but longer (monthly median range: 125–286 m, Table S7), ETEs between October and March and more frequent (monthly median range: 0.11–0.167 Table S6), but shorter (monthly median range: 76–90 m Table S7), ETEs between April and September. This suggests a trade-off between ETE frequency and ETE distance, and potentially a negative correlation. Yet, when we investigated this relationship further, we found a positive correlation between ETE frequency and ETE distance (Pearson’s product-moment correlation: t = 21.667, df = 3705, p-value < 2.2e-16) (Figure S3). The positive correlation appears to be driven by male badgers (Figure S4), as the correlation varied seasonally for female badgers (Figure S5). Because we saw variation in the correlation of ETE frequency and ETE distance across sexes and seasons, we developed a third way of considering ETEs; an ETE ‘investment’ metric (the product of ETE frequency and distance – results from GLMM provided in Table S8). This metric allowed an estimate of the investment by badgers in extraterritorial activity across the year.

Overall, female badgers made both shorter (Fig. 5) and less frequent (Fig. 4) ETEs than males, especially between late autumn and late spring (October to March). Consequently, female badgers appeared to invest less in ETEs than male badgers throughout the year (Figure S6). In addition, it appeared that both males (Figure S7) and females (Figure S8) invested more in ETEs during the second half of the year. The strongest peak in ETE investment by female badgers was by older females in November (Figure S8). By contrast, both juvenile and adult males showed minor peaks in ETE investment in spring, and much larger peaks later in the year (Figure S7).

## Discussion

Our data suggest that ETEs are the rule in, rather than the exception to, badger behaviour. The majority of collared badgers (>50% of collared badger population) in our study made ETEs in all months and all badgers made ETEs during the month of September.

We found seasonal patterns to the number of badgers making ETEs, the frequency with which badgers made ETEs and the extent that ETEs encroached into neighbouring territories.

Badgers made less frequent (post-hoc tests, Table S9), but longer (post-hoc tests, Table S10), ETEs between April and September and more frequent, but shorter, ETEs between October and March.

Differences between higher density populations and our medium density one may help explain the motivation of ETEs. Woodroffe *et al*.^28^ found peaks in the frequency of ETEs by badgers in February and September. While all of our study badgers made ETEs in September, corresponding with the September peak found by Woodroffe *et al*.^28^, such correspondence was not present in February. In February only 59% of female badgers (10 out of 17) and 94% of male badgers (16 out of 17) in our study made ETEs (Fig. 2). Consequently, in our population, there was no peak in the frequency of ETEs in February.

ETEs clearly provide opportunities for badgers to encounter and mate with members of social groups other than their own^9,10^. If this is the main motivation behind ETEs, then we would expect the seasonal peaks in ETE activity to match seasonal peaks in mating activity. Badgers exhibit peaks in mating activity in February and April/May^34^. These peaks coincide with peaks in the circulating level of testosterone^8^ (Table S11). We see peaks in the ETE ‘investment’ metric by males in February, but also between September and November (Figure S4). So, while higher levels of circulating testosterone may explain higher ETE investment by males in winter/spring, it does not explain their higher ETE investment in autumn (Table S11).

Seasonal peaks in badger ETEs have also been linked to the oestrus cycle^28^. In an Irish study^11^, female badgers were found in oestrus throughout the year, but the proportion in each month varied widely, with peaks in February, May/June, August and November. The three months of highest ETE ‘investment’ by female badgers (Figure S5) coincide with the oestrus peaks in Irish badgers (Table S11). In addition, some oestrus peaks coincide with peaks in male ETE investment. Despite the apparent importance of circulating testosterone and oestrus state in explaining ETE investment, the addition of such data did not improve the performance of models for either male or female ETE activity.

ETEs have been suggested to facilitate foraging at resource-rich patches in neighbouring territories^8^. However, if ETEs were primarily for food gathering, we would expect to see them more frequently in summer, when food is less abundant, and less frequently in spring and autumn, when food is plentiful^35,36^. The steady pattern of shorter, but more frequent, ETEs between April and September does not suggest a change in ETE behaviour during summer. This indicates that the ETEs of our study badgers were not motivated by food resources.

Badgers probably receive continuously updated information about the composition of neighbouring social groups from the olfactory signals deposited at boundary latrines. Indeed, latrines may provide detailed information about familiar (i.e. social group members), neighbouring and unfamiliar individuals^37,38^ to badgers. In other carnivore species, communal defecation sites can convey information about social status and reproductive condition^39,40^. It is possible that ETEs serve a monitoring role, additional to that obtained by boundary latrine visits. Badgers were investing energy in visiting neighbouring territories during the summer months (Tables S8 and S9), but it is unclear whether this involved visits to hinterland latrines^8,41^ or contact with members of neighbouring social groups. Badger activity is higher during the summer months^28,42^ and home ranges are larger^43^ (approximating territory size). Journeys into neighbouring territories at that time of year are more likely to result in an encounter with a member of a neighbouring social group than would be the case in winter months, when badger activity is lower and home ranges are much smaller than territory sizes. We know that up to 100% of tracked badgers can engage in inter-social group mixing in a given week, during spring and summer^44^, although data from Irish studies suggest this mixing may be much less frequent in medium-density populations^32^. Despite this disparity, we propose that an additional purpose of ETEs, especially the shorter, ‘summer’ ETEs which we observed, may be to increase the chance of direct encounters with members of neighbouring social groups., or indirect encounters, such as the detection of scent marks.

Such constant monitoring of neighbouring populations may explain why ‘missing’ badgers (either dead or dispersed) are quickly replaced by members of neighbouring social groups. While scent marks serve as proxies for the presence of territory owners^45,46^, their dissipation will indicate the absence of territory owners. In our study population we regularly saw ‘replacement’ badgers arrive within a week of the disappearance of a resident badger. This suggests that badger scent signals dissipate in a similar way to those of red foxes^46^ and other mustelid species^47^. And, although a great deal of olfactory information is available at border latrines, badgers may be making regular ETEs simply to confirm the presence of individuals within a neighbouring group.

There was a difference in the seasonal patterns of ETEs made by male and female badgers. We found that, on average, female badgers made their longest ETEs in January (Fig. 5). Females give birth to cubs between mid-January and mid-March^11,34^ (Table S11) and the presence of non-breeding females in a social group has been demonstrated to have negative effects on the physical condition of breeding females^48^. Therefore, it is likely that breeding females are aggressive towards non-breeding females during pregnancy and while suckling cubs^8,30^. This may prompt non-breeding females to explore dispersal opportunities. Although our dataset excluded those badgers which were in the process of dispersing successfully (see Methods), long ETEs may have been produced by unsuccessful dispersal attempts. This idea is supported by the finding that most dispersing females begin their dispersal in January or February^6^. While relatively few females made ETEs in January (Fig. 2), those that did make them, made them infrequently (Fig. 4) and tended to go far from home (annual median ETE = 87 m, Jan median = 370 m) (Fig. 5).

Both male and female badgers made long ETEs (Fig. 5), and showed high ETE investment, in November (Figure S6). This is just before the beginning of winter lethargy, a period when there is a substantial drop in the frequency of the daily ranging activity of both male and female badgers^42^. November is at the end of the autumn breeding season^8^ and may represent the last opportunity for badgers to mate before females implant blastocysts and begin pregnancy^8,11^. While the frequency of ETEs diminished in November (Fig. 2), both male and female badgers were making much longer ETEs than normal (Males: annual median ETE = 131 m, Nov median = 314 m; Females: annual median ETE = 87 m, Nov median = 263 m). This shows that badgers (both male and female) were encroaching further into neighbouring social groups during November and investing more time and energy into ETE activity (Table S12). It is likely they were seeking extra-group matings.

Rather than an increase in territorial behaviour during the autumn mating season, our data provide evidence of an increase in the distance of incursions made into neighbouring territories (and potentially beyond). This appears to be a strategy to allow outbreeding, and is much more in keeping with a “kleptogamic strategy” (badgers stealing matings outside their social group) than the anti-kleptogamic strategy (resident males attempting to prevent neighbours from gaining reproductive access to resident females) reported by Roper *et al*.^49^.

Females give birth to cubs between mid-January and mid-March^11,34^. While we found that, on average, female badgers made their longest ETEs in January (Fig. 5), we believe these long ETEs were made by non-breeding females making unsuccessful dispersal attempts, as breeding females are aggressive towards non-breeding females during pregnancy and while suckling cubs^8,30^. This idea is supported by the finding that most dispersing females begin their dispersal in January or February^6^. While relatively few females made ETEs in January (Fig. 2), those that did make them, made them infrequently (Fig. 4) and tended to go far from home (annual median ETE = 87 m, Jan median = 370 m) (Fig. 5). Although we might have expected breeding females to make few, if any, ETEs during pregnancy, and while cubs were very young, breeding females did make ETEs throughout winter and spring (Table S13). Perhaps for this reason, and despite an anticipated contrast in behaviour, we were unable to detect a difference between the ETE distance, frequency or investment of breeding and non-breeding females in January (Table S13).

In our medium density population (1.8 badgers km^−2^), we would expect fewer non-breeding females than in the higher density populations (5–6 badgers km^−2^) of southwest England^50,51^. Indeed, the number of non-breeding females in our study was further reduced by plural breeding in several social groups (Table S14). We found that non-breeding females made more frequent ETEs than breeding females in March and April (Figure S9). This may have been due to pre-dispersal prospecting in the non-breeding females^52^. It therefore seems likely that the February peak in ETE activity seen in southwest England^28^, but not in our study, may be due to a higher number of non-breeding females in the English population.

Females showed their greatest investment in ETEs in November (Figure S6, Table S12). Within November, it was the older females which invested most heavily in ETEs (Figure S8). Since male badgers tend to disperse over shorter distances^6^, older females were likely to be related to many of the neighbouring males. To avoid inbreeding, the older females would, therefore, have needed to undertake longer ETEs to find less closely-related males. An additional investigation of female badgers revealed that it was breeding females (recorded as lactating at spring capture) which were making greater efforts to undertake ETEs just before the start of winter lethargy (Table S13). As most adult females conceive in spring or early summer and most fertilised eggs survive the period of delayed implantation^8^, it appears that breeding females were making efforts to find more distant males in November. Such a strategy may maximise outbreeding.

The potential for frequent contacts between neighbouring social groups may give cause for concern regarding the transmission of TB^53^. However, we found that the ETE activity of cubs was different from older badgers. For example, over 50% of cubs made no ETEs during the months they wore collars (October, November and December). Furthermore, cubs made relatively short incursions (mean = 174 m, median = 129 m, max = 734 m), while yearlings travelled much further into their neighbours’ territories (mean = 338 m, median = 157 m, max = 4232 m). It would appear advantageous, therefore, to vaccinate badgers against TB while they are cubs, and before they begin making ETEs into neighbouring social groups. This would minimise the potential for TB transmission during ETEs. In addition, if vaccination were completed prior to September (a peak in adult ETE activity – Fig. 2), this would have the benefit of protecting cubs against TB transmission from visiting adults.

Several territorial mammals exhibit pre-dispersal prospecting^20,21,26,52,54,55^. While we excluded badgers, which were later found to be in the process of dispersing, from this analysis (see Methods), we did see evidence of pre-dispersal prospecting by badgers in our study area^6^. Since successful dispersers were excluded from our analyses, any pre-dispersal prospecting which was captured in this study must have been made by badgers which were unsuccessful in their dispersal attempts. Despite the inclusion of such movements, our dataset represents the activity of badgers which remained faithful to their ‘home’ social group territories.

It is important to emphasise that ETEs were only recorded when badgers encroached more than 15 m into a neighbouring territory, as the manufacturers of the GPS collars reported a 15 m accuracy to GPS fixes^56^. This means that visits to boundary latrines and their immediate vicinity were not recorded as ETEs. While every care was taken to do so, if the territories of the badgers were not accurately described (see Methods) it is possible that some within-territory movements may have been identified as short ETEs. Similarly, some ETEs may have been ascribed as within-territory movements. However, as all mean ETE distances for male and female cohorts were over 100 m, such concerns would probably have little influence on the reported results.

To adjust to changing ecological circumstances (both biotic and abiotic), most animals attempt to gather information to reduce uncertainty^57^. Even apparent errors in behaviour could be intentional information gathering^58^. However, gathering any information requires trading off time and energy which might otherwise be expended on basic biological demands, such as growth and reproduction. Despite the associated costs, information use is a key feature of adaptive behaviour and central to organismal biology^59^. While researchers in cognitive biology have recently embraced this idea, it does not yet appear to have gained currency in mammal ecology.

In summary, our data show that badgers of both sexes make ETEs throughout the year. During a large part of the year, badgers made frequent, but short, incursions into their neighbours’ territories. This may allow social groups to monitor the composition and health of their neighbours. Seasonal peaks in ETE ‘investment’, by both male and female badgers, generally coincide with peaks of circulating sex hormones. This is consistent with badgers seeking extra-group mating opportunities, to allow outbreeding. As cubs make relatively few, short ETEs, it appears most practical to target this age cohort with any vaccination programmes. If vaccination could be completed before September, this would have the additional benefit of protecting cubs against the visits of potentially infected adults.

## Materials & Methods

### Study area

The study was conducted in Co. Wicklow, Ireland, around the site of a national road upgrade and realignment^60^. We identified the local population density of badgers to be 1.8 badgers km^−2^, using a capture frequency technique^61^. The study area was a matrix of undulating agricultural land (75%), with patches of mixed and coniferous woodland (14%) with small residential areas and farmyards scattered throughout (7%). Local farming practices include pasture (cattle and sheep), arable (primarily wheat, barley and maize) and some equestrian activity. In any given year, 68.5% of agricultural land, on average, was under pasture, while at least 16.7% was arable crops. A well-developed hedgerow system connected fields.

### Ethical statement

This project was approved by Trinity College Dublin’s Animal Research Ethics Committee (Project No. 290516) and the Health Products Regulatory Authority (Project No. 7024754). Badgers were captured in accordance with licences (NPWS Nos. 101/2009, 04/2010, 13/2010, C123/2010, 03/2011, C040/2011, C03/2013, C005/2013 and C001/2015) as required by the Wildlife Act, 1976.

### Trapping and handling of badgers

Badgers were captured in two trapping events per annum: April-May (3–4 weeks) and September-October (3–4 weeks) using cage traps (following the methods in Cheeseman & Mallinson 1980) and, on occasions when necessary, stopped-restraints with a minimum closure of 32.5 cm (Wildlife Act, 1976, Regulations 2003 (S.I. 620 of 2003)^62^;). Cage traps were of a standard DAFM approved design, 1.1 m to 1.3 m long, about 35 cm wide and 35 cm high, and were constructed from 3 cm square 8 gauge galvanised mesh, hot dipped and finished in a smooth black plastic coating (Rathcormac Steel Supplies, Rathcormac, Co. Sligo, Ireland). The triggering mechanism was by means of a string trip-line, the breaking of which closed the trap door behind the badger. Both cage traps and stopped-restraints conformed to national legislation for humane trapping defined in the Wildlife Act, 1976, Regulations 2003 (S.I. 620 of 2003). Stopped-restraints were used at setts where no badgers had been caught in cages and where badger activity was evident indicating the presence of “cage-shy” badgers (cages 97%, stopped-restraints 3% of captures). Cages were baited with peanuts for two weeks prior to and during the trapping event. All fieldwork was carried out with the land owners’ consent. Data collection began in April 2010 and continued until August 2016.

Captured badgers were anaesthetised in-cage by veterinary practitioners from DAFM using ketamine hydrochloride (Narketan 10® or Vetalar®) at 10 mg/kg and medetomidine (Domitor® or Medetor®) at 0.1 mg/kg^63^. This dose was delivered by intramuscular injection into the lumbar muscles using a pole syringe.

Age was determined by the dentition^64,65^ and general appearance of each badger. Individuals in their first year were defined as cubs, as yearlings in their second year and as adults in their third year or over. Sex was determined by external examination.

All badgers were marked by an implanted Radio Frequency Identification (RFID) microchip and a tattoo, on first capture. The last four digits of the microchip number were tattooed to the right medial thigh (inside hindleg). The tattoo and microchip numbers were used to identify individual animals at subsequent recaptures during the study. Badgers were vaccinated against TB with Bacille Calmette-Guérin (BCG) vaccine by intramuscular injection into the lumbar muscles. All badgers were weighed, clinically examined for signs of ill-health, external wounds and parasite load, and records taken. Blood samples and pharyngeal swabs were taken to determine TB infection status (BrockTB Stat-Pak®) and selective mycobacterial culture, respectively.

### Data Collection

We aimed to capture as many badgers as possible within each social group. Badgers weighing 8 kg or more and with a suitable neck to head ratio (that is, a cranial circumference of at least 1 cm more than the neck circumference) were fitted with a Tellus Light GPS collar (Followit Wildlife, Lindesberg, Sweden). This meant that collars weighed no more than 3% of a badger’s body weight. Such criteria usually precluded the collaring of cubs, except perhaps during autumn trapping events when they were heavy enough to wear a collar. If badgers were recaptured in the same trapping session, they were identified (RFID), recorded and released without anaesthetic. If badgers were caught while wearing collars from a previous season, those collars were replaced to ensure sufficient battery life for the new season. Collars were programmed to take four GPS readings per night at 10 pm, 11 pm, 1am and 2am, when badgers were expected to be above ground, except in April, May and September when 8 readings a night where taken hourly between 9 pm and 4am^60^. More frequent readings were timed to coincide with subsequent trapping sessions, to maximise the likelihood of trapping badgers which were wearing collars. In order to equalise sampling effort across the year, only four GPS readings per night (10 pm, 11 pm, 1am and 2am) were included from the data collected in April, May and September. This provided us with a maximum of four GPS readings per night, across the year, for any given badger. Over the course of the study 139 badgers were trapped and 83 of those were fitted with collars.

### Identifying ETEs

In order to calculate the length and frequency of ETEs, the boundaries of social groups needed to be determined. As GPS data was accumulated it became clear that an earlier bait-marking study of the area did not accurately correspond to territory boundaries in the study population. Because badgers reach maximum ranging in summer^28,66–68^, in areas where social groups are contiguous, the shape of their summer ranges should reflect the physical borders between social groups. For each social group in each year, 95% Minimum Convex Polygons (MCPs) were calculated using the summer GPS locations, i.e. June, July and August, of all of the collared members of the social group (summer MCPs). Where we did not have data for badgers in June, July and August (because batteries may have failed or badgers were not trapped until the autumn trapping session) we included all available GPS data for social group members in that calendar year. However, because MCPs are convex, they can include areas that are never used and/or belong to other social groups, particularly when territories are irregularly shaped. Therefore, they may also include GPS locations that were actually ETEs. While this is not a problem when using MCP estimates as a proxy for home range size, it poses a difficultly in determining when a GPS location is within a territory boundary or outside it.

In order to map realistic territory boundaries, and to decide objectively which GPS locations constituted ETEs, summer MCPs were plotted in ArcMap (ESRI, Redlands, CA, USA) along with all contributing GPS locations. A ‘geographical territory boundary’ was digitised based on the location of a summer MCP polygon, the real linear landscape features in the study area (roads, hedgerows, rivers and observed badger-paths)^69^ and the GPS locations of the relevant social group. The boundaries for each year were compared and only changed if it was very clear that members of the social group were ranging differently from the years before/after (Table S1). Most social groups were relatively stable. However, there were some examples of fission or fusion of social groups^68,70,71^, and the building of the M11 altered the position of some territory boundaries adjacent to the road^43^ (Table S1).

As the territorial boundaries in our study area were contiguous (Fig. 1), it was possible to describe any ETE in two possible ways: (i) an ETE was a journey made by a badger beyond the territorial limits of its ‘home’ social group, or, (ii) an ETE was a journey made by a badger inside the territorial limits of a different social group.

To determine ETE distances, the ‘Generate Near Table’ tool in ArcMap was used to calculate the distance (m) between each of a badger’s GPS points and the nearest edge of the polygon representing the boundary of their social group in that year. All points that fell outside the polygon returned a distance of >0 m and could be considered ETEs. As the Followit GPS collar locations could be inaccurate by up to 15 m^56^, only locations that were >15 m from the polygon where identified as ETEs. On a given night, a badger may have recorded more than one GPS location outside its territory boundary. In such cases, only the GPS location that was furthest from the badger’s territory boundary was retained in the dataset. The frequency of ETEs (fETE) for each badger in any month (described as a proportion between 0 and 1) was calculated by dividing the number of active nights in a month by the number of ETEs made in that month. Active nights were defined as the number of nights in a month in which that the badger was above ground for long enough to record GPS locations and wore a working collar. This meant that fETE values could vary from 0.032 (one ETE in a 31-day month) to 1.0 (an ETE during every day of the month).

Badgers that had been identified as super-rangers^43^ or were actively engaged in dispersal were excluded from the ETE dataset. During dispersal, badgers change their ‘home’ social group. This is reflected by their movements centring on a new social group. Potentially, such movements may appear to include a great many ETEs. However, until the badger has settled in its new social group, it is not clear where ‘home’ is, and so it is not possible to assess which movements are ETEs. The exclusion of super-rangers and dispersing badgers resulted in 838 records of monthly ETE frequency for badgers, 198 of which were zeroes (i.e. no ETEs in that month) and an ETE distance dataset of 4164 records.

### ETE ‘investment’ metric

This metric was calculated by multiplying the ETE frequency (a metric calculated across an entire month) of a given badger by its longest ETE on any given day. This potentially resulted in daily estimates of ETE investment for each badger. The resultant data were analysed in a GLMM (see below).

### Data analysis

The data were analysed in three ways: the number of badgers undertaking ETEs each month, the frequency per month with which they went on ETEs and the distance they travelled when on ETEs. Frequency of ETEs was estimated for all badgers, even those which did not make ETEs. As a result, the frequency of ETE dataset was zero-inflated. Datasets of the frequency and distance of ETEs were explored for differences between the sexes and ages of the badgers, and the month of the year in which the ETE occurred.

All analyses were performed in R (v3.6.1)^72^. Both year and the individual identity of badgers (nested within their social groups) were used as random variables within the GLMMs, to prevent pseudoreplication (Tables S15 and S16). As the ETE frequency dataset was zero-inflated, we performed the GLMM analyses of these data within the glmmTMB package^73^, allowing for overdispersal within the dataset by setting dispformula = ~1. ETE distance data and ETE ‘investment’ data were log-transformed prior to analysis, to allow for the log-normal distribution of the raw data. As the transformed data produced overdispersed models, they were also analysed within the glmmTMB package. We started with saturated models (Tables S15 and S16) and compared the relative performance of subsidiary models with their AICc values using the dredge function from the MuMIn package^74^.

## Supplementary information

Supplementary Information.

## Data Availability

All the data analysed in this study are available at Zenodo, 10.5281/zenodo.3560733.

## References

[1] Kruuk H (1978). Foraging and spatial-organization of European badger, Meles meles L. Behavioral Ecology and Sociobiology.

[2] Kruuk H (1978). Spatial organization and territorial behavior of European badger Meles meles. Journal of Zoology.

[3] Pope LC, Domingo-Roura X, Erven K, Burke T (2006). Isolation by distance and gene flow in the Eurasian badger (Meles meles) at both a local and broad scale. Molecular Ecology.

[4] Rogers LM (1998). Movement of badgers (Meles meles) in a high-density population: individual, population and disease effects. Proceedings of the Royal Society B-Biological Sciences.

[5] Huck M, Frantz AC, Dawson DA, Burke T, Roper TJ (2008). Low genetic variability, female-biased dispersal and high movement rates in an urban population of Eurasian badgers Meles meles. Journal of Animal Ecology.

[6] Gaughran, A. *et al*. Dispersal patterns in a medium-density Irish badger population: Implications for understanding the dynamics of tuberculosis transmission. *Ecology and Evolution*, 10.1002/ece3.5753 (2019). 10.1002/ece3.5753 PMC6912907 31871635

[7] Christian SF (1994). Dispersal and other intergroup movements in badgers, Meles meles. Zeitschrift Fur Saugetierkunde-International Journal of Mammalian Biology.

[8] Roper, T. J. Badger. (HarperCollins, 2010).

[9] Carpenter PJ (2005). Mating system of the Eurasian badger, Meles meles, in a high density population. Molecular Ecology.

[10] Dugdale HL, Macdonald DW, Pope LC, Burke T (2007). Polygynandry, extra-group paternity and multiple-paternity litters in European badger (Meles meles) social groups. Molecular Ecology.

[11] Corner, L. A. L., Stuart, L. J., Kelly, D. J. & Marples, N. M. Reproductive Biology Including Evidence for Superfetation in the European Badger Meles meles (Carnivora: Mustelidae). *Plos One* **10**, 10.1371/journal.pone.0138093 (2015). 10.1371/journal.pone.0138093 PMC4605486 26465324

[12] Greenwood PJ (1980). Mating systems, philopatry and dispersal in birds and mammals. Animal Behaviour.

[13] Keller LF, Arcese P, Smith JNM, Hochachka WM, Stearns SC (1994). Selection against inbred song sparrows during a natural population bottleneck. Nature.

[14] Huisman J, Kruuk LEB, Ellis PA, Clutton-Brock T, Pemberton JM (2016). Inbreeding depression across the lifespan in a wild mammal population. Proceedings of the National Academy of Sciences of the United States of America.

[15] O’Brien, S. J. *et al*. Genetic basis for species vulnerability in an endangered species the African cheetah. *Science*, **53**, 10.1126/science.2983425 (1988).

[16] Pusey A, Wolf M (1996). Inbreeding avoidance in animals. Trends in Ecology &. Evolution.

[17] Archie EA (2007). Behavioural inbreeding avoidance in wild African elephants. Molecular Ecology.

[18] Sparkman AM, Adams JR, Steury TD, Waits LP, Murray DL (2012). Pack social dynamics and inbreeding avoidance in the cooperatively breeding red wolf. Behavioral Ecology.

[19] Woodroffe R, Macdonald DW, da Silva J (1993). Dispersal and philopatry in the European badger, Meles meles. Journal of Zoology.

[20] Mayer, M., Zedrosser, A. & Rosell, F. Extra-territorial movements differ between territory holders and subordinates in a large, monogamous rodent. *Scientific Reports* **7**, 10.1038/s41598-017-15540-0 (2017). 10.1038/s41598-017-15540-0 PMC5681683 29127395

[21] Havens RP, Crawford JC, Nelson TA (2013). Survival, Home Range, and Colony Reproduction of Beavers in East-Central Illinois, an Agricultural Landscape. American Midland Naturalist.

[22] Young AJ, Spong G, Clutton-Brock T (2007). Subordinate male meerkats prospect for extra-group paternity: alternative reproductive tactics in a cooperative mammal. Proceedings of the Royal Society B-Biological Sciences.

[23] Soulsbury CD, Iossa G, Baker PJ, White PCL, Harris S (2011). Behavioral and spatial analysis of extraterritorial movements in red foxes (Vulpes vulpes). Journal of Mammalogy.

[24] Rioux, M. J., Lai, S., Casajus, N., Bety, J. & Berteaux, D. Winter home range fidelity and extraterritorial movements of Arctic fox pairs in the Canadian High Arctic. *Polar Research* **36**, 10.1080/17518369.2017.1316930 (2017).

[25] White, P. J., Ralls, K. & Siniff, D. B. Nocturnal encounters between kit foxes. *Journal of Mammalogy* **81**, 456–461, doi:10.1644/1545-1542(2000)081<0456:nebkf>2.0.co;2 (2000).

[26] Mancinelli S, Ciucci P (2018). Beyond home: Preliminary data on wolf extraterritorial forays and dispersal in Central Italy. Mammalian Biology.

[27] Macdonald DW, Newman C, Buesching CD, Johnson PJ (2008). Male-biased movement in a high-density population of the Eurasian badger (Meles meles). Journal of Mammalogy.

[28] Woodroffe R (2017). Ranging behaviour of badgers Meles meles vaccinated with Bacillus Calmette Guerin. Journal of Applied Ecology.

[29] Silk MJ (2017). Seasonal variation in daily patterns of social contacts in the European badger Meles meles. Ecology and Evolution.

[30] Kruuk, H. *The social badger: ecology and behaviour of a group-living carnivore (Meles meles)* (Oxford University Press, 1989).

[31] Sleeman, D. P. & Mulcahy, M. F. In The Badger (ed Hayden T & Murray D) 154–165 (Royal Irish Academy, 1993).

[32] O’Mahony DTB (2015). Meles meles) contact metrics in a medium-density population. Mammalian Biology.

[33] Byrne AW (2014). Large-scale movements in European badgers: has the tail of the movement kernel been underestimated?. Journal of Animal Ecology.

[34] Neal, E. G. & Cheeseman, C. Badgers. (T and AD Poyser Ltd, 1998).

[35] Cleary GP, Corner LAL, O’Keeffe J, Marples NM (2009). The diet of the badger Meles meles in the Republic of Ireland. Mammalian Biology.

[36] Cleary GP, Corner LAL, O’Keeffe J, Marples NM (2011). Diet of the European badger (Meles meles) in the Republic of Ireland: A comparison of results from an analysis of stomach contents and rectal faeces. Mammalian Biology.

[37] Buesching CD, Waterhouse JS, Macdonald DW (2002). Gas-chromatographic analyses of the subcaudal gland secretion of the European badger (Meles meles) Part I: Chemical differences related to individual parameters. Journal of Chemical Ecology.

[38] Palphramand KL, White PCL (2007). Badgers, Meles meles, discriminate between neighbour, alien and self scent. Animal Behaviour.

[39] Gorman, M. L. & Trowbridge, B. J. in Carnivore Behavior, Ecology, and Evolution (ed Gittleman J. L.) 57–88 (Springer, 1989).

[40] Tinnesand, H. V. *et al*. Will trespassers be prosecuted or assessed according to their merits? A consilient interpretation of territoriality in a group-living carnivore, the European badger (Meles meles). *PLoS ONE* **10**, 10.1371/journal.pone.0132432 (2015). 10.1371/journal.pone.0132432 PMC4493095 26147753

[41] Kilshaw K, Newman C, Buesching C, Bunyan J, Macdonald D (2009). Coordinated latrine use by European badgers, Meles meles: potential consequences for territory defense. Journal of Mammalogy.

[42] Kowalczyk R, Jedrzejewska B, Zalewski A (2003). Annual and circadian activity patterns of badgers (Meles meles) in Bialowieza Primeval Forest (eastern Poland) compared with other Palaearctic populations. Journal of Biogeography.

[43] Gaughran, A. *et al*. Super-ranging. A new ranging strategy in European badgers. *Plos One* **13**, 10.1371/journal.pone.0191818 (2018). 10.1371/journal.pone.0191818 PMC5812585 29444100

[44] Ellwood SA (2017). An active-radio-frequency-identification system capable of identifying co-locations and social-structure: Validation with a wild free-ranging animal. Methods in Ecology and Evolution.

[45] Kruuk H (1992). Scent marking by otters (Lutra lutra) - signaling the use of resources. Behavioral Ecology.

[46] Potts JR, Harris S, Giuggioli L (2013). Quantifying behavioral changes in territorial animals caused by sudden population declines. American Naturalist.

[47] Rasa OA (1973). Marking behaviour and its social significance in the African dwarf mongoose, Helogale undulata rufula. Z. Tierpsychol..

[48] Woodroffe R, Macdonald DW (2000). Helpers provide no detectable benefits in the European badger (Meles meles). Journal of Zoology.

[49] Roper TJ, Shepherdson DJ, Davies JM (1986). Scent marking with faeces and anal secretion in the European badger (Meles meles) - seasonal and spatial characteristics of latrine use in relation to territoriality. Behaviour.

[50] Judge, J., Wilson, G. J., Macarthur, R., Delahay, R. J. & McDonald, R. A. Density and abundance of badger social groups in England and Wales in 2011-2013. *Scientific Reports* **4**, 10.1038/srep03809 (2014). 10.1038/srep03809 PMC3899851 24457532

[51] Woodroffe R (2016). Badgers prefer cattle pasture but avoid cattle: implications for bovine tuberculosis control. Ecology Letters.

[52] Roper TJ, Ostler JR, Conradt L (2003). The process of dispersal in badgers Meles meles. Mammal Review.

[53] Weber N (2013). Badger social networks correlate with tuberculosis infection. Current Biology.

[54] Doolan SP, Macdonald DW (1996). Dispersal and extra-territorial prospecting by slender-tailed meerkats (Suricata suricatta) in the south-western Kalahari. Journal of Zoology.

[55] Mulder JL (1985). S-organization, movements and dispersal in a Dutch red fox (Vulpes vulpes) population - some preliminary results. Revue D’Ecologie - La Terre et La Vie.

[56] Mullen EM (2015). The avoidance of farmyards by European badgers Metes meles in a medium density population. Applied Animal Behaviour Science.

[57] Dall SRX, Giraldeau LA, Olsson O, McNamara JM, Stephens DW (2005). Information and its use by animals in evolutionary ecology. Trends in Ecology & Evolution.

[58] Marples NM, Speed MP, Thomas RJ (2018). An individual-based profitability spectrum for understanding interactions between predators and their prey. Biological Journal of the Linnean Society.

[59] Danchin E, Giraldeau LA, Valone TJ, Wagner RH (2004). Public information: From nosy neighbors to cultural evolution. Science.

[60] MacWhite T, Maher P, Mullen E, Marples N, Good M (2013). Satellite tracking study of badgers (Meles meles) to establish normal ranging behaviour prior to a road realignment. Irish Naturalists’ Journal.

[61] Chao A (1988). Estimating animal abundance with capture frequency data. Journal of Wildlife Management.

[62] Murphy D, O’Keeffe JJ, Martin SW, Gormley E, Corner LAL (2009). An assessment of injury to European badgers (Meles meles) due to capture in stopped restraints. Journal of Wildlife Diseases.

[63] Davison KE (2007). Evaluation of the anaesthetic effects of combinations of ketamine, medetomidine, romifidine and butorphanol in European badgers (Meles meles). Veterinary Anaesthesia and Analgesia.

[64] da Silva J, Macdonald DW (1989). Limitations to the use of tooth wear as a means of aging Eurasian badgers, Meles meles. Revue D Ecologie-La Terre Et La Vie.

[65] Hancox M (1988). A review of age determination criteria in the Eurasian badger. Lynx (Praha).

[66] Kauhala K (1995). Changes in distribution of the European badger Meles meles in Finland during the rapid colonization of the raccoon dog. Annales Zoologici Fennici.

[67] Kowalczyk R, Zalewski A, Jedrzejewska B (2006). Daily movement and territory use by badgers Meles meles in Bialowieza Primeval Forest, Poland. Wildlife Biology.

[68] Revilla E, Palomares F (2002). Spatial organization, group living and ecological correlates in low-density populations of Eurasian badgers, Meles meles. Journal of Animal Ecology.

[69] O’Brien J, Elliott S, Hayden TJ (2016). Use of hedgerows as a key element of badger (Meles meles) behaviour in Ireland. Mammalian Biology.

[70] da Silva J, Woodroffe R, Macdonald DW (1993). Habitat, food availability and group territoriality in the European badger, Meles meles. Oecologia.

[71] van Apeldoorn RC, Vink J, Matyastik T (2006). Dynamics of a local badger (Meles meles) population in the Netherlands over the years 1983–2001. Mammalian Biology.

[72] R: A language and environment for statistical computing v. 3.6.1 (2019-07-05)–“Action of the Toes” (R Foundation for Statistical Computing, Vienna, Austria, 2019).

[73] Brooks ME (2017). glmmTMB Balances Speed and Flexibility Among Packages for Zero-inflated Generalized Linear Mixed Modeling. R Journal.

[74] MuMIn: multi-model inference. v. 1.42.1. (2018).

