# Supplementary material for: Extra Territorial Excursions by European badgers are not limited by age, sex or season
Source: Sci Rep. 2020 Jun 15;10:9665. doi: 10.1038/s41598-020-66809-w (PMC7296015; doi:10.1038/s41598-020-66809-w)
Supplement: Supplementary file 1 — Supplementary Information. [file 41598_2020_66809_MOESM1_ESM.pdf]

Extra Territorial Excursions by European badgers are not limited by age, sex or season.

David J. Kelly1*, Aoibheann Gaughran1, Enda Mullen2, Teresa MacWhite3, Peter Maher3, Margaret Good4 and Nicola M. Marples1

Supplementary Information

Figure S1. Plot to show the sex-biased differences for ETE distance. Female badgers are indicated with a pink shaded area, Male badgers are indicated with a blue shaded area.

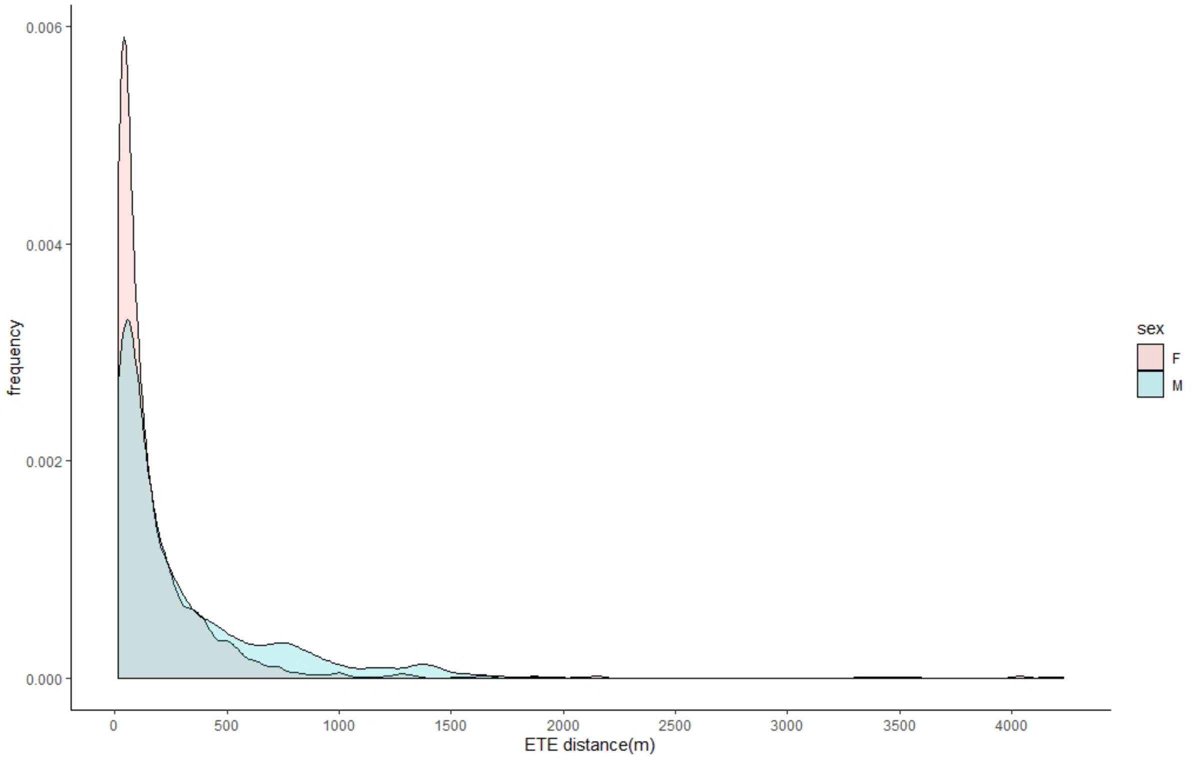

Figure S2. Plot to show the age-biased differences, within the *Juvenile* cohort. Cubs are indicated with a pink shaded area, Yearlings are indicated with a blue shaded area.

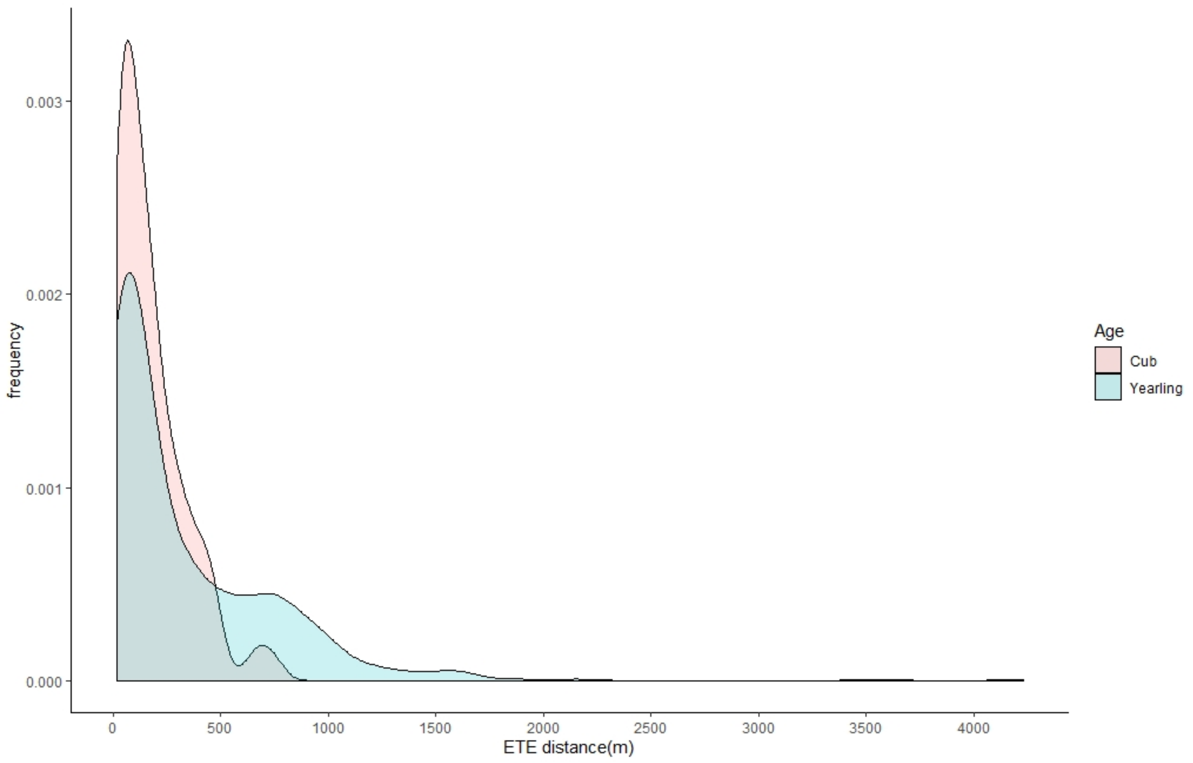

Figure S3. Scatterplot displaying the correlation between the frequency of ETEs and the log of the distance of those ETEs.

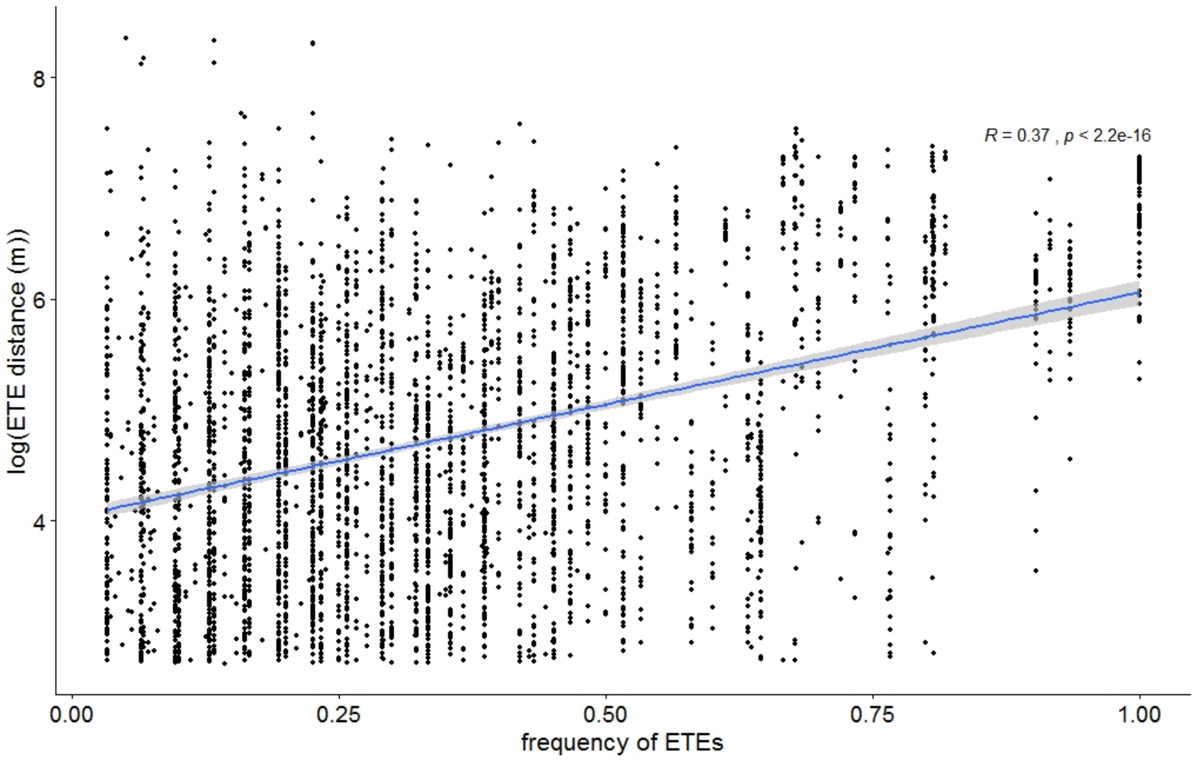

Figure S4. A combined plot of the relationship between ETE frequency and ETE distance for **male** badgers across the year, from A) January to L) December. Lines of best fit for the correlations (in blue) are provided for each month, along with the variance (grey shaded area), the correlation coefficient and P value from a Pearson's product-moment correlation test.
NB Correlation coefficients are positive for all months.

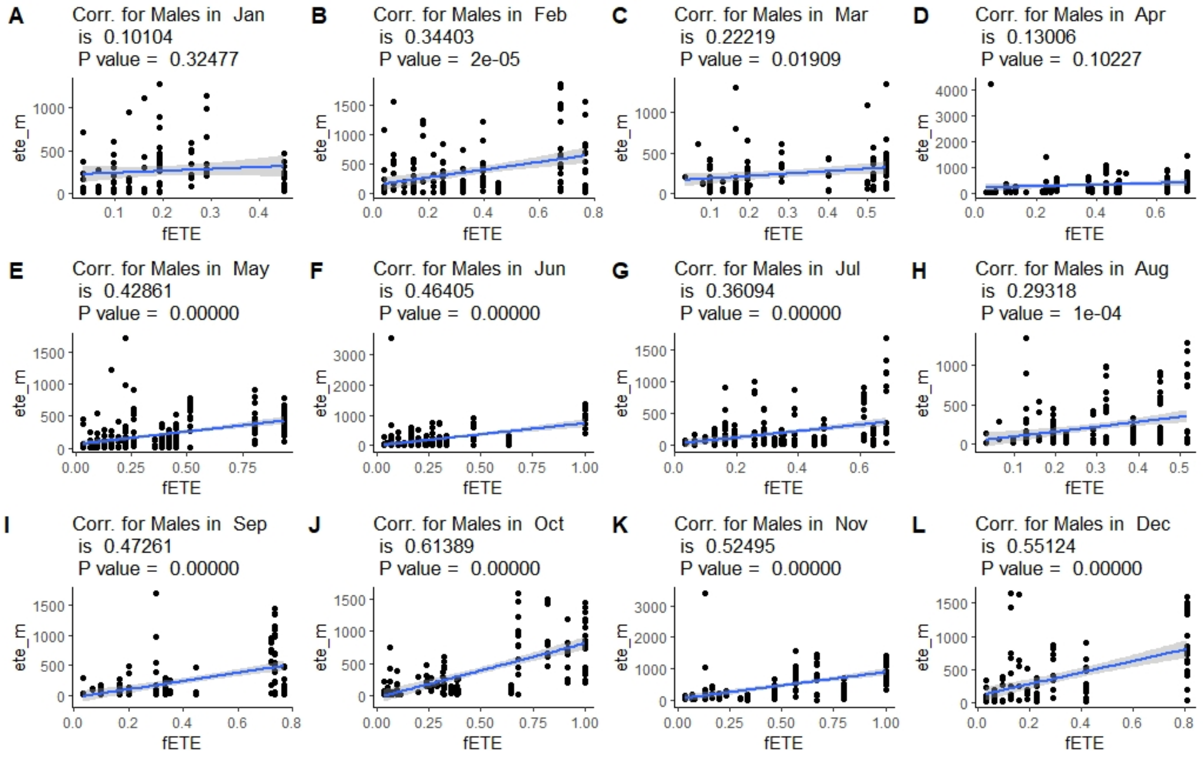

Figure S5. A combined plot of the relationship between ETE frequency and ETE distance for **female** badgers across the year, from A) January to L) December. Lines of best fit for the correlations (in blue) are provided for each month, along with the variance (grey shaded area), the correlation coefficient and P value from a Pearson's product-moment correlation test.
NB Correlation coefficients are **NOT** positive for all months.

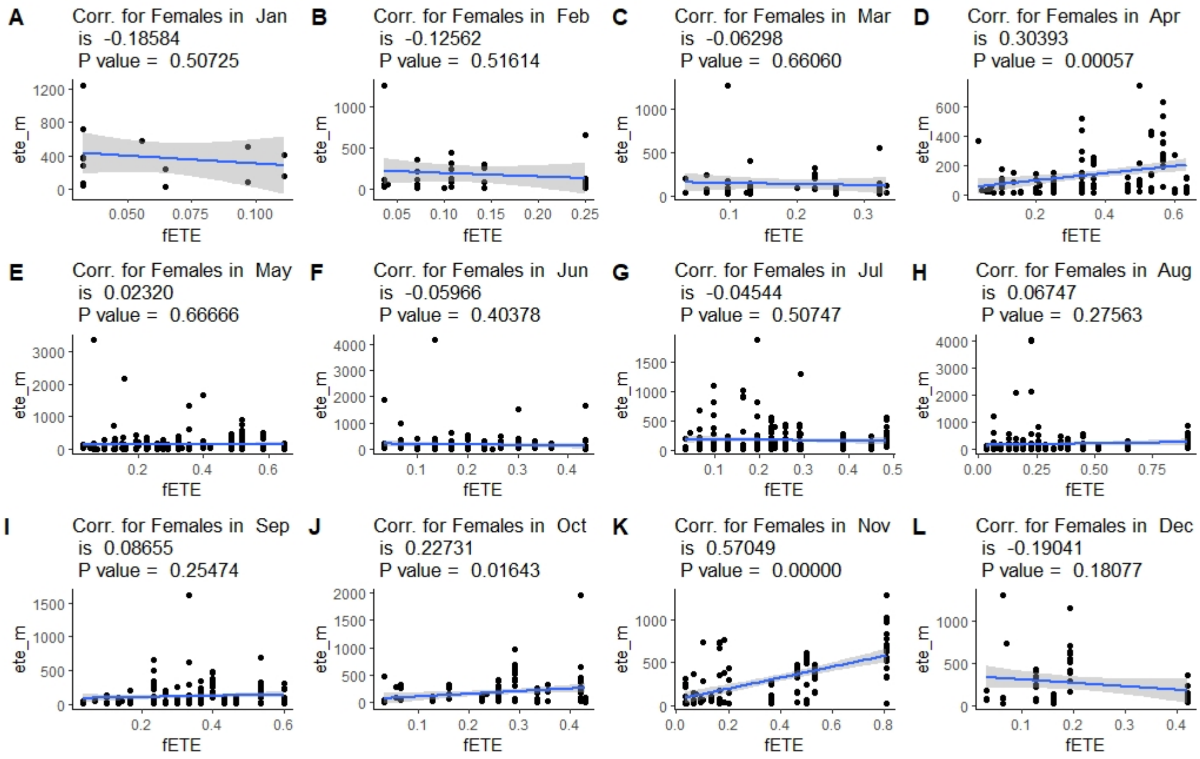

Figure S6. **ETE investment** demonstrated by male (M.month) (empty boxes) and female (F.month) (grey boxes) badgers across the year. Boxes represent the second and third quartiles, while bars within these boxes represent median values. Whiskers extend up to 1.5 times the interquartile range (Q3 – Q1) from the boxes. Data points outside the range of the whiskers are represented individually by empty open circles. Grey dots mark the means of each sex/month cohort.

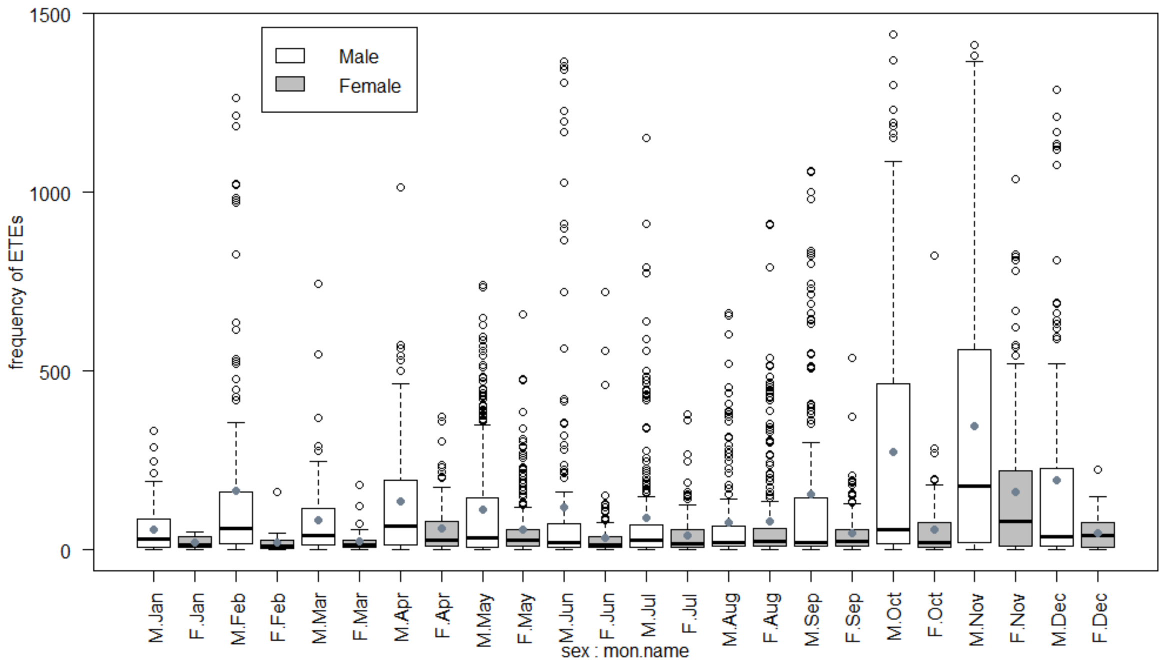

Figure S7. Boxplot of the **ETE investment metric** (ETE distance * ETE frequency) for three age classes (Juveniles – pale blue bars, Young Adults – darker blue bars, Older Adults – grey bars) of **male badgers** across the year. Boxes represent the second and third quartiles of the data, while bars within these boxes represent median values. Whiskers extend up to 1.5 times the interquartile range (Q3 – Q1) from the boxes. Data points outside the range of the whiskers are represented individually by empty circles.

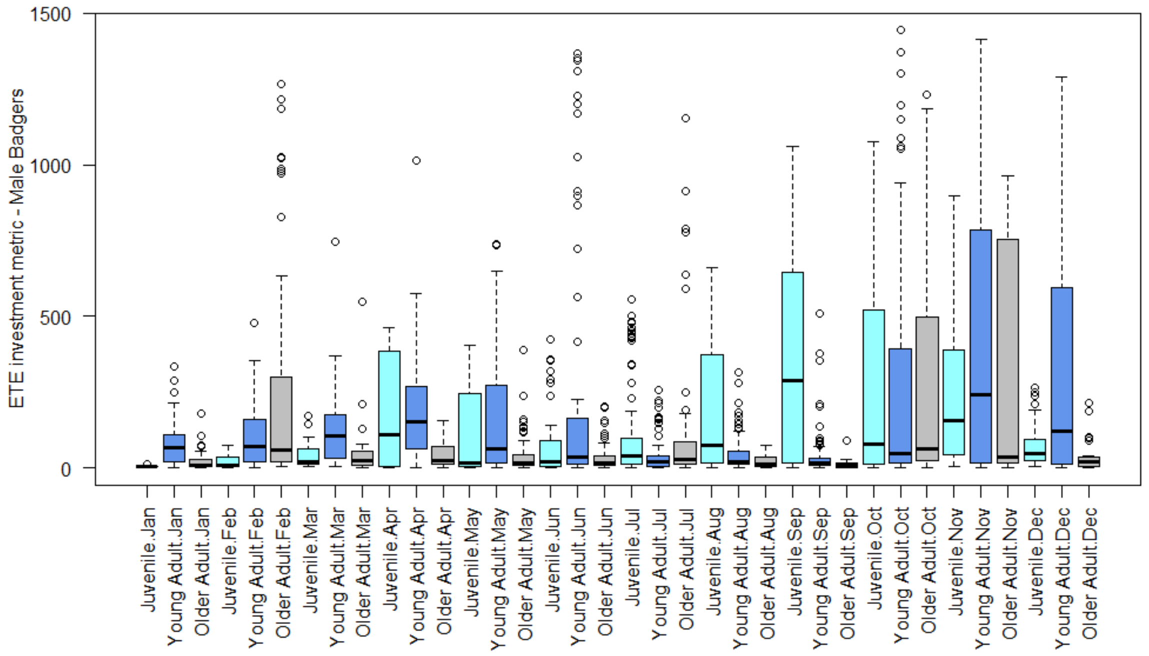

Figure S8. Boxplot of the **ETE investment metric** (ETE distance * ETE frequency) for three age classes (Juveniles – pale blue bars, Young Adults – darker blue bars, Older Adults – grey bars) of **female badgers** across the year. Boxes represent the second and third quartiles of the data, while bars within these boxes represent median values. Whiskers extend up to 1.5 times the interquartile range (Q3 – Q1) from the boxes. Data points outside the range of the whiskers are represented individually by empty circles.

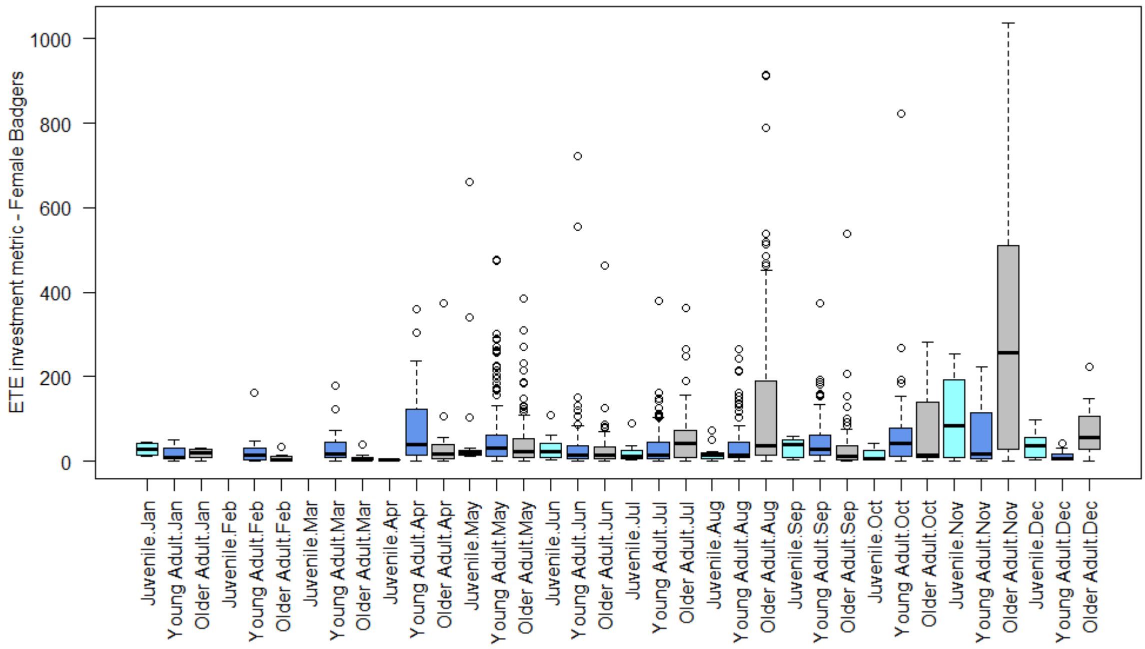

Figure S9. Plot to show the difference in ETE frequency between **breeding** (showing signs of lactation at spring capture) (white bars labelled YES.Month) and **non-breeding** (grey bars labelled NO.Month) **female badgers** across the year.

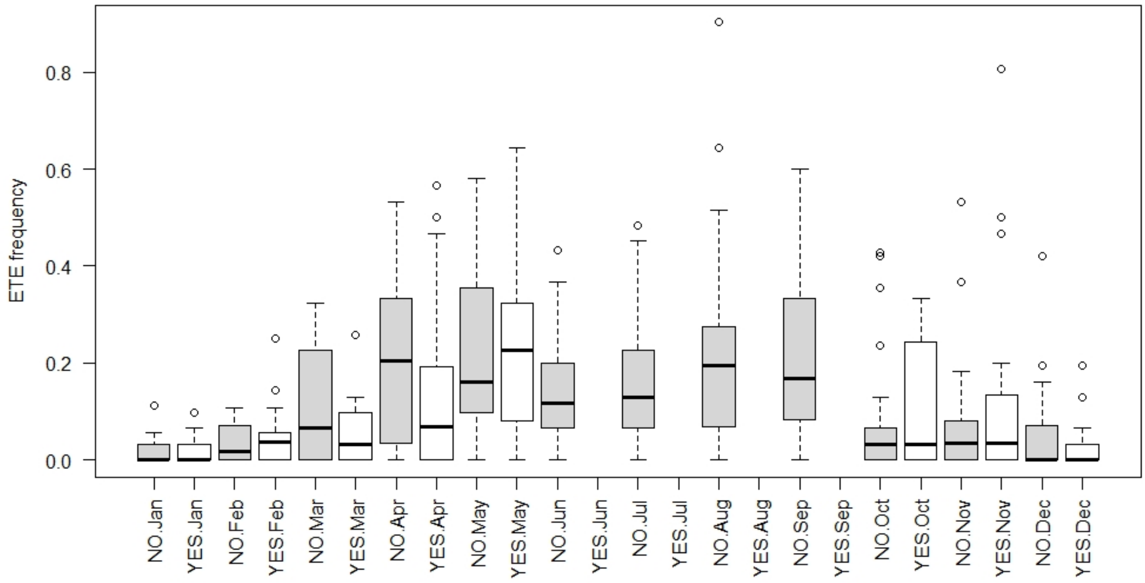

Table S1. Social group sizes (km^2) across the study period. Cells with underlined values indicate changes in territory size/shape due to fission/fusion processes.

Social Group	2010	2011	2012	2013	2014	2015	2016
Pines	1.10	1.42	1.44	1.78	1.78	2.14	2.11
Hawthorn	2.07	2.76	2.49	1.02	0.75	0.79	0.96
Quarry		2.31	2.95	2.50	2.65	1.89	1.93
Bracken	1.85	1.92	1.38	2.57	2.12	2.34	2.61
Oak		1.52	1.69	1.70	1.68	1.48	1.50
Dogwood	1.84	1.21	1.43	2.26	2.02	2.05	2.06
Big Tree			1.00	2.71	2.78	2.82	1.04
Driving Range				1.26	1.24	1.25	1.69
Orchard				0.87	0.99	1.09	1.01
Cemetery	0.74	0.52	1.09	0.68	*	1.32	1.47
Bluebell	1.60	1.47	1.11				
Lodge							1.77
Sycamore		1.10	0.81	1.26	3.51	3.33	2.79
Briars	0.75	1.04	*	1.98		1.32	

Table S2. Output from emmeans (Lenth 2018) post-hoc tests for age cohort (as an independent term) within the best model explaining variation in **ETE frequency.**

> emmeans(my.model, pairwise ~ cohort, adjust="holm")

$`emmeans`

cohort	emmean	SE	df	lower.CL	upper.CL
Juvenile	0. 127	0. 0315	819	0.0654	0. 189
Young Adult	0. 192	0. 0297	819	0.1339	0. 250
Older Adult	0.150	0.0311	819	0.0887	0.211

Results are averaged over the levels of: mon.name, sex
Confidence level used: 0.95

$contrasts

contrast	estimate	SE	df	t.ratio	p.value	
Juvenile - Young Adult	-0.0649	0.0180	819	-3.609	0.0010	**
Juvenile - Older Adult	-0.0225	0.0237	819	-0.950	0.3426	NS
Young Adult - Older Adult	0.0424	0.0171	819	2.477	0.0269	*

Results are averaged over the levels of: mon.name, sex
P value adjustment: holm method for 3 tests

Table S3. Output from emmeans (Lenth 2018) post-hoc tests for the month:sex interaction within the saturated model for **ETE frequency.**

> emmeans(my.model, pairwise ~ month | sex, adjust="holm")

$`emmeans`

sex = F:

month	emmean	SE	df	lower.CL	upper.CL
Jan	0.0284	0.0387	783	-0.047549	0.104
Feb	0.0277	0.0452	783	-0.060928	0.116
Mar	0.0934	0.0473	783	0.000571	0.186
Apr	0.1646	0.0415	783	0.083204	0.246
May	0.2098	0.0362	783	0.138824	0.281
Jun	0.1365	0.036	783	0.065877	0.207
Jul	0.1492	0.036	783	0.07852	0.22
Aug	0.1788	0.0369	783	0.106382	0.251
Sep	0.2183	0.0406	783	0.138525	0.298
Oct	0.0884	0.035	783	0.019644	0.157
Nov	0.103	0.0344	783	0.035425	0.171
Dec	0.0457	0.0357	783	-0.024415	0.116

sex = M:

month	emmean	SE	df	lower.CL	upper.CL
Jan	0.0885	0.0379	783	0.014027	0.163
Feb	0.1667	0.0388	783	0.090607	0.243
Mar	0.1507	0.0399	783	0.072409	0.229
Apr	0.2423	0.0393	783	0.165168	0.319
May	0.2204	0.035	783	0.151633	0.289
Jun	0.2011	0.0356	783	0.131215	0.271
Jul	0.2274	0.0361	783	0.156547	0.298
Aug	0.1998	0.0379	783	0.125326	0.274
Sep	0.2577	0.0404	783	0.178362	0.337
Oct	0.1818	0.0366	783	0.109917	0.254
Nov	0.173	0.0365	783	0.101293	0.245
Dec	0.1101	0.0364	783	0.03868	0.182

Results are averaged over the levels of: cohort3
Confidence level used: 0.95

$contrasts
sex = F:

contrast	estimate	SE	df	t.ratio	p.value	
Jan – Feb	0.000682	0.0424	783	0.016	1.0000	
Jan – Mar	-0.064976	0.0446	783	-1.456	1.0000	
Jan – Apr	-0.136222	0.0389	783	-3.502	0.0269	*
Jan – May	-0.181419	0.0341	783	-5.315	<.0001	***
Jan – Jun	-0.108069	0.0341	783	-3.173	0.0785	
Jan – Jul	-0.120828	0.0347	783	-3.486	0.0280	*
Jan – Aug	-0.150432	0.0356	783	-4.229	0.0016	***
Jan – Sep	-0.189898	0.0397	783	-4.780	0.0001	***
Jan – Oct	-0.059986	0.0343	783	-1.751	1.0000	
Jan – Nov	-0.074637	0.0336	783	-2.219	1.0000	
Jan – Dec	-0.017318	0.0347	783	-0.499	1.0000	
Feb – Mar	-0.065658	0.0487	783	-1.347	1.0000	
Feb – Apr	-0.136904	0.0442	783	-3.100	0.0961	
Feb – May	-0.182101	0.0407	783	-4.471	0.0006	***
Feb – Jun	-0.108751	0.0408	783	-2.662	0.3404	
Feb – Jul	-0.121510	0.0415	783	-2.929	0.1645	
Feb – Aug	-0.151114	0.0423	783	-3.574	0.0209	*
Feb – Sep	-0.190579	0.0457	783	-4.173	0.0020	***
Feb – Oct	-0.060668	0.0416	783	-1.460	1.0000	
Feb – Nov	-0.075319	0.0411	783	-1.831	1.0000	
Feb – Dec	-0.018000	0.0421	783	-0.427	1.0000	
Mar – Apr	-0.071246	0.0463	783	-1.539	1.0000	
Mar – May	-0.116443	0.0430	783	-2.708	0.3044	
Mar – Jun	-0.043093	0.0431	783	-1.000	1.0000	
Mar – Jul	-0.055852	0.0437	783	-1.278	1.0000	
Mar – Aug	-0.085456	0.0445	783	-1.921	1.0000	
Mar – Sep	-0.124921	0.0478	783	-2.613	0.3843	
Mar – Oct	0.004991	0.0439	783	0.114	1.0000	
Mar – Nov	-0.009661	0.0435	783	-0.222	1.0000	
Mar – Dec	0.047658	0.0444	783	1.073	1.0000	
Apr – May	-0.045197	0.0363	783	-1.245	1.0000	
Apr – Jun	0.028153	0.0365	783	0.772	1.0000	
Apr – Jul	0.015394	0.0373	783	0.413	1.0000	
Apr – Aug	-0.014210	0.0381	783	-0.373	1.0000	
Apr – Sep	-0.053676	0.0417	783	-1.288	1.0000	
Apr – Oct	0.076236	0.0372	783	2.049	1.0000	
Apr – Nov	0.061585	0.0368	783	1.673	1.0000	
Apr – Dec	0.118904	0.0379	783	3.138	0.0864	
May – Jun	0.073350	0.0301	783	2.435	0.6204	
May – Jul	0.060591	0.0309	783	1.958	1.0000	
May – Aug	0.030987	0.0319	783	0.970	1.0000	
May – Sep	-0.008478	0.0366	783	-0.232	1.0000	
May – Oct	0.121433	0.0316	783	3.839	0.0077	***
May – Nov	0.106782	0.0313	783	3.411	0.0361	*
May – Dec	0.164101	0.0326	783	5.026	<.0001	***
Jun – Jul	-0.012759	0.0302	783	-0.423	1.0000	
Jun – Aug	-0.042363	0.0314	783	-1.349	1.0000	

Jun – Sep	-0.081828	0.0363	783	-2.253	0.9569	
Jun – Oct	0.048084	0.0314	783	1.534	1.0000	
Jun – Nov	0.033432	0.0310	783	1.079	1.0000	
Jun – Dec	0.090751	0.0324	783	2.805	0.2329	
Jul – Aug	-0.029604	0.0314	783	-0.941	1.0000	
Jul – Sep	-0.069069	0.0362	783	-1.907	1.0000	
Jul – Oct	0.060843	0.0313	783	1.946	1.0000	
Jul – Nov	0.046191	0.0310	783	1.492	1.0000	
Jul – Dec	0.103510	0.0324	783	3.199	0.0732	
Aug – Sep	-0.039465	0.0369	783	-1.070	1.0000	
Aug – Oct	0.090447	0.0322	783	2.811	0.2329	
Aug – Nov	0.075795	0.0319	783	2.373	0.7151	
Aug – Dec	0.133114	0.0333	783	3.996	0.0042	**
Sep – Oct	0.129912	0.0359	783	3.616	0.0182	**
Sep – Nov	0.115261	0.0358	783	3.223	0.0687	
Sep – Dec	0.172579	0.0370	783	4.663	0.0002	***
Oct – Nov	-0.014651	0.0288	783	-0.508	1.0000	
Oct – Dec	0.042667	0.0302	783	1.415	1.0000	
Nov – Dec	0.057319	0.0293	783	1.953	1.0000	

sex = M:

contrast	estimate	SE	df	t.ratio	p.value	
Jan – Feb	-0.078266	0.0334	783	-2.340	0.9486	
Jan – Mar	-0.062250	0.0349	783	-1.785	1.0000	
Jan – Apr	-0.153845	0.0346	783	-4.445	0.0007	***
Jan – May	-0.131930	0.0311	783	-4.238	0.0016	**
Jan – Jun	-0.112605	0.0318	783	-3.544	0.0242	*
Jan – Jul	-0.138978	0.0331	783	-4.201	0.0019	**
Jan – Aug	-0.111356	0.0350	783	-3.181	0.0870	
Jan – Sep	-0.169278	0.0379	783	-4.467	0.0006	***
Jan – Oct	-0.093344	0.0333	783	-2.801	0.2869	
Jan – Nov	-0.084515	0.0333	783	-2.540	0.5870	
Jan – Dec	-0.021628	0.0327	783	-0.661	1.0000	
Feb – Mar	0.016016	0.0355	783	0.451	1.0000	
Feb – Apr	-0.075579	0.0353	783	-2.143	1.0000	
Feb – May	-0.053664	0.0319	783	-1.683	1.0000	
Feb – Jun	-0.034339	0.0325	783	-1.057	1.0000	
Feb – Jul	-0.060712	0.0338	783	-1.795	1.0000	
Feb – Aug	-0.033090	0.0358	783	-0.926	1.0000	
Feb – Sep	-0.091012	0.0386	783	-2.359	0.9288	
Feb – Oct	-0.015078	0.0340	783	-0.443	1.0000	
Feb – Nov	-0.006249	0.0341	783	-0.183	1.0000	
Feb – Dec	0.056638	0.0335	783	1.688	1.0000	
Mar – Apr	-0.091595	0.0364	783	-2.518	0.6115	
Mar – May	-0.069680	0.0331	783	-2.107	1.0000	
Mar – Jun	-0.050355	0.0337	783	-1.496	1.0000	
Mar – Jul	-0.076728	0.0350	783	-2.189	1.0000	
Mar – Aug	-0.049106	0.0369	783	-1.330	1.0000	
Mar – Sep	-0.107028	0.0397	783	-2.698	0.3843	
Mar – Oct	-0.031094	0.0354	783	-0.879	1.0000	
Mar – Nov	-0.022265	0.0353	783	-0.630	1.0000	

Mar – Dec	0.040622	0.0349	783	1.163	1.0000	
Apr – May	0.021916	0.0321	783	0.683	1.0000	
Apr – Jun	0.041240	0.0329	783	1.253	1.0000	
Apr – Jul	0.014867	0.0343	783	0.433	1.0000	
Apr – Aug	0.042489	0.0362	783	1.173	1.0000	
Apr – Sep	-0.015433	0.0391	783	-0.395	1.0000	
Apr – Oct	0.060501	0.0348	783	1.736	1.0000	
Apr – Nov	0.069330	0.0348	783	1.994	1.0000	
Apr – Dec	0.132217	0.0344	783	3.843	0.0080	**
May – Jun	0.019325	0.0280	783	0.689	1.0000	
May – Jul	-0.007048	0.0295	783	-0.239	1.0000	
May – Aug	0.020573	0.0318	783	0.647	1.0000	
May – Sep	-0.037348	0.0353	783	-1.058	1.0000	
May – Oct	0.038586	0.0309	783	1.249	1.0000	
May – Nov	0.047415	0.0309	783	1.535	1.0000	
May – Dec	0.110302	0.0305	783	3.613	0.0190	*
Jun – Jul	-0.026373	0.0301	783	-0.877	1.0000	
Jun – Aug	0.001249	0.0324	783	0.039	1.0000	
Jun – Sep	-0.056673	0.0357	783	-1.586	1.0000	
Jun – Oct	0.019261	0.0315	783	0.612	1.0000	
Jun – Nov	0.028090	0.0315	783	0.891	1.0000	
Jun – Dec	0.090977	0.0311	783	2.921	0.2012	
Jul – Aug	0.027622	0.0326	783	0.847	1.0000	
Jul – Sep	-0.030300	0.0358	783	-0.846	1.0000	
Jul – Oct	0.045634	0.0317	783	1.439	1.0000	
Jul – Nov	0.054463	0.0318	783	1.710	1.0000	
Jul – Dec	0.117350	0.0316	783	3.719	0.0129	*
Aug – Sep	-0.057921	0.0371	783	-1.561	1.0000	
Aug – Oct	0.018012	0.0336	783	0.535	1.0000	
Aug – Nov	0.026841	0.0338	783	0.794	1.0000	
Aug – Dec	0.089728	0.0335	783	2.675	0.4047	
Sep – Oct	0.075934	0.0361	783	2.105	1.0000	
Sep – Nov	0.084763	0.0362	783	2.343	0.9486	
Sep – Dec	0.147650	0.0359	783	4.109	0.0027	**
Oct – Nov	0.008829	0.0314	783	0.281	1.0000	
Oct – Dec	0.071716	0.0310	783	2.311	0.9914	
Nov – Dec	0.062887	0.0306	783	2.053	1.0000	

Results are averaged over the levels of: cohort3
P value adjustment: holm method for 66 tests

Table S4. Output from emmeans (Lenth 2018) post-hoc tests for the month:sex interaction within the best model explaining variation in **ETE distance.**

> emmeans(mymodel, pairwise ~ month | sex, adjust="holm")
>
$`emmeans`

sex = F:

**month**	**emmean**	**SE**	**df**	**lower.CL**	**upper.CL**
Jan	5.88	0.2839	3673	5.32	6.44
Feb	4.85	0.2157	3673	4.43	5.28
Mar	4.64	0.1747	3673	4.3	4.98
Apr	4.52	0.1251	3673	4.28	4.77
May	4.58	0.0979	3673	4.39	4.77
Jun	4.65	0.1119	3673	4.43	4.87
Jul	4.68	0.1096	3673	4.47	4.9
Aug	4.56	0.105	3673	4.36	4.77
Sep	4.47	0.1149	3673	4.24	4.69
Oct	4.76	0.1311	3673	4.5	5.01
Nov	5.05	0.1267	3673	4.8	5.3
Dec	5.21	0.1774	3673	4.86	5.55

sex = M:

**mon.name**	**emmean**	**SE**	**df**	**lower.CL**	**upper.CL**
Jan	5.45	0.1419	3673	5.17	5.72
Feb	5.56	0.1235	3673	5.32	5.8
Mar	5.37	0.131	3673	5.11	5.62
Apr	5.1	0.1175	3673	4.87	5.33
May	4.77	0.1049	3673	4.56	4.97
Jun	4.82	0.1093	3673	4.61	5.04
Jul	4.67	0.1071	3673	4.46	4.88
Aug	4.82	0.1152	3673	4.59	5.04
Sep	4.32	0.1165	3673	4.1	4.55
Oct	4.87	0.1183	3673	4.64	5.1
Nov	5.11	0.119	3673	4.87	5.34
Dec	5.35	0.1327	3673	5.09	5.61

Results are averaged over the levels of: cohort3
Results are given on the log (not the response) scale.
Confidence level used: 0.95

$contrasts

sex = F:

contrast	estimate	SE	df	t.ratio	p.value	
Jan–Feb	1.02743	0.3288	3673	3.124	0.0898	
Jan–Mar	1.24100	0.3070	3673	4.042	0.0031	**
Jan–Apr	1.35648	0.2889	3673	4.695	0.0002	***
Jan–May	1.29963	0.2799	3673	4.643	0.0002	***
Jan–Jun	1.22987	0.2818	3673	4.365	0.0008	***
Jan–Jul	1.19777	0.2809	3673	4.264	0.0012	**
Jan–Aug	1.31785	0.2813	3673	4.685	0.0002	***
Jan–Sep	1.41052	0.2852	3673	4.946	0.0001	***
Jan–Oct	1.12396	0.2896	3673	3.881	0.0058	**
Jan–Nov	0.82950	0.2908	3673	2.853	0.1960	
Jan–Dec	0.67385	0.3099	3673	2.175	1.0000	
Feb–Mar	0.21356	0.2386	3673	0.895	1.0000	
Feb–Apr	0.32905	0.2174	3673	1.513	1.0000	
Feb–May	0.27220	0.2074	3673	1.312	1.0000	
Feb–Jun	0.20244	0.2110	3673	0.959	1.0000	
Feb–Jul	0.17034	0.2099	3673	0.811	1.0000	
Feb–Aug	0.29041	0.2104	3673	1.380	1.0000	
Feb–Sep	0.38309	0.2149	3673	1.783	1.0000	
Feb–Oct	0.09653	0.2205	3673	0.438	1.0000	
Feb–Nov	-0.19794	0.2239	3673	-0.884	1.0000	
Feb–Dec	-0.35358	0.2518	3673	-1.404	1.0000	
Mar–Apr	0.11549	0.1766	3673	0.654	1.0000	
Mar–May	0.05864	0.1654	3673	0.355	1.0000	
Mar–Jun	-0.01112	0.1705	3673	-0.065	1.0000	
Mar–Jul	-0.04322	0.1696	3673	-0.255	1.0000	
Mar–Aug	0.07685	0.1696	3673	0.453	1.0000	
Mar–Sep	0.16953	0.1753	3673	0.967	1.0000	
Mar–Oct	-0.11704	0.1842	3673	-0.635	1.0000	
Mar–Nov	-0.41150	0.1867	3673	-2.204	1.0000	
Mar–Dec	-0.56714	0.2218	3673	-2.557	0.4666	
Apr–May	-0.05685	0.1100	3673	-0.517	1.0000	
Apr–Jun	-0.12661	0.1243	3673	-1.019	1.0000	
Apr–Jul	-0.15871	0.1223	3673	-1.297	1.0000	
Apr–Aug	-0.03864	0.1187	3673	-0.325	1.0000	
Apr–Sep	0.05404	0.1252	3673	0.432	1.0000	
Apr–Oct	-0.23252	0.1415	3673	-1.643	1.0000	
Apr–Nov	-0.52698	0.1397	3673	-3.772	0.0089	**
Apr–Dec	-0.68263	0.1868	3673	-3.654	0.0136	*
May–Jun	-0.06976	0.0953	3673	-0.732	1.0000	
May–Jul	-0.10186	0.0932	3673	-1.093	1.0000	
May–Aug	0.01821	0.0884	3673	0.206	1.0000	
May–Sep	0.11089	0.0996	3673	1.114	1.0000	
May–Oct	-0.17567	0.1205	3673	-1.458	1.0000	
May–Nov	-0.47014	0.1183	3673	-3.976	0.0040	**
May–Dec	-0.62578	0.1721	3673	-3.636	0.0143	*
Jun–Jul	-0.03210	0.1018	3673	-0.315	1.0000	
Jun–Aug	0.08797	0.1013	3673	0.869	1.0000	
Jun–Sep	0.18065	0.1126	3673	1.605	1.0000	

Jun–Oct	-0.10591	0.1286	3673	-0.823	1.0000	
Jun–Nov	-0.40037	0.1294	3673	-3.095	0.0953	
Jun–Dec	-0.55602	0.1780	3673	-3.124	0.0898	
Jul–Aug	0.12007	0.0965	3673	1.244	1.0000	
Jul–Sep	0.21275	0.1096	3673	1.942	1.0000	
Jul–Oct	-0.07381	0.1266	3673	-0.583	1.0000	
Jul–Nov	-0.36827	0.1265	3673	-2.911	0.1665	
Jul–Dec	-0.52392	0.1764	3673	-2.969	0.1412	
Aug–Sep	0.09268	0.1050	3673	0.882	1.0000	
Aug–Oct	-0.19389	0.1254	3673	-1.546	1.0000	
Aug–Nov	-0.48835	0.1224	3673	-3.989	0.0039	**
Aug–Dec	-0.64399	0.1759	3673	-3.661	0.0135	*
Sep–Oct	-0.28656	0.1300	3673	-2.204	1.0000	
Sep–Nov	-0.58103	0.1309	3673	-4.437	0.0006	***
Sep–Dec	-0.73667	0.1820	3673	-4.049	0.0031	**
Oct–Nov	-0.29446	0.1412	3673	-2.085	1.0000	
Oct–Dec	-0.45011	0.1862	3673	-2.417	0.6750	
Nov–Dec	-0.15564	0.1772	3673	-0.879	1.0000	

sex = M:

contrast	estimate	SE	df	t.ratio	p.value	
Jan–Feb	-0.11613	0.1389	3673	-0.836	1.0000	
Jan–Mar	0.08029	0.1456	3673	0.552	1.0000	
Jan–Apr	0.34394	0.1439	3673	2.390	0.4391	
Jan–May	0.67737	0.1349	3673	5.020	<.0001	***
Jan–Jun	0.62088	0.1400	3673	4.434	0.0004	***
Jan–Jul	0.77451	0.1373	3673	5.643	<.0001	***
Jan–Aug	0.62932	0.1406	3673	4.477	0.0004	***
Jan–Sep	1.12080	0.1452	3673	7.719	<.0001	***
Jan–Oct	0.57479	0.1442	3673	3.987	0.0027	**
Jan–Nov	0.33926	0.1452	3673	2.336	0.4693	
Jan–Dec	0.09864	0.1471	3673	0.670	1.0000	
Feb–Mar	0.19642	0.1317	3673	1.492	1.0000	
Feb–Apr	0.46007	0.1241	3673	3.708	0.0076	**
Feb–May	0.79350	0.1147	3673	6.918	<.0001	***
Feb–Jun	0.73701	0.1216	3673	6.061	<.0001	***
Feb–Jul	0.89064	0.1174	3673	7.585	<.0001	***
Feb–Aug	0.74545	0.1239	3673	6.018	<.0001	***
Feb–Sep	1.23692	0.1273	3673	9.715	<.0001	***
Feb–Oct	0.69092	0.1272	3673	5.432	<.0001	***
Feb–Nov	0.45538	0.1281	3673	3.556	0.0126	*
Feb–Dec	0.21477	0.1359	3673	1.581	1.0000	
Mar–Apr	0.26365	0.1302	3673	2.026	0.9863	
Mar–May	0.59708	0.1207	3673	4.947	<.0001	***
Mar–Jun	0.54059	0.1283	3673	4.214	0.0011	**
Mar–Jul	0.69422	0.1246	3673	5.573	<.0001	***
Mar–Aug	0.54903	0.1298	3673	4.230	0.0011	**
Mar–Sep	1.04050	0.1329	3673	7.829	<.0001	***
Mar–Oct	0.49450	0.1353	3673	3.655	0.0091	**
Mar–Nov	0.25896	0.1343	3673	1.929	1.0000	
Mar–Dec	0.01835	0.1411	3673	0.130	1.0000	

Apr–May	0.33343	0.1049	3673	3.180	0.0475	*
Apr–Jun	0.27694	0.1144	3673	2.421	0.4365	
Apr–Jul	0.43057	0.1104	3673	3.899	0.0037	**
Apr–Aug	0.28538	0.1173	3673	2.432	0.4365	
Apr–Sep	0.77685	0.1190	3673	6.526	<.0001	***
Apr–Oct	0.23085	0.1241	3673	1.860	1.0000	
Apr–Nov	-0.00469	0.1230	3673	-0.038	1.0000	
Apr–Dec	-0.24530	0.1355	3673	-1.811	1.0000	
May–Jun	-0.05649	0.0984	3673	-0.574	1.0000	
May–Jul	0.09714	0.0963	3673	1.009	1.0000	
May–Aug	-0.04805	0.1030	3673	-0.466	1.0000	
May–Sep	0.44343	0.1078	3673	4.113	0.0017	**
May–Oct	-0.10258	0.1133	3673	-0.906	1.0000	
May–Nov	-0.33811	0.1111	3673	-3.044	0.0706	
May–Dec	-0.57873	0.1250	3673	-4.629	0.0002	***
Jun–Jul	0.15363	0.1020	3673	1.507	1.0000	
Jun–Aug	0.00844	0.1098	3673	0.077	1.0000	
Jun–Sep	0.49992	0.1154	3673	4.334	0.0007	***
Jun–Oct	-0.04609	0.1206	3673	-0.382	1.0000	
Jun–Nov	-0.28162	0.1192	3673	-2.363	0.4539	
Jun–Dec	-0.52224	0.1308	3673	-3.992	0.0027	**
Jul–Aug	-0.14519	0.1058	3673	-1.373	1.0000	
Jul–Sep	0.34629	0.1092	3673	3.171	0.0475	*
Jul–Oct	-0.19972	0.1147	3673	-1.741	1.0000	
Jul–Nov	-0.43525	0.1139	3673	-3.821	0.0050	**
Jul–Dec	-0.67587	0.1274	3673	-5.305	<.0001	***
Aug–Sep	0.49147	0.1149	3673	4.279	0.0009	***
Aug–Oct	-0.05453	0.1209	3673	-0.451	1.0000	
Aug–Nov	-0.29007	0.1197	3673	-2.424	0.4365	
Aug–Dec	-0.53068	0.1313	3673	-4.042	0.0022	**
Sep–Oct	-0.54601	0.1194	3673	-4.575	0.0002	***
Sep–Nov	-0.78154	0.1193	3673	-6.552	<.0001	***
Sep–Dec	-1.02216	0.1348	3673	-7.580	<.0001	***
Oct–Nov	-0.23553	0.1170	3673	-2.013	0.9863	
Oct–Dec	-0.47615	0.1332	3673	-3.576	0.0120	*
Nov–Dec	-0.24062	0.1277	3673	-1.885	1.0000	

Results are averaged over the levels of: cohort3
Results are given on the log (not the response) scale.
P value adjustment: holm method for 66 tests

Table S5. Summary of age cohorts (as an independent term) within the best model explaining variation in **ETE distance.**

```
> with(my.data, tapply(ETE.distance, list("Age Cohort#"=age.cohort), mean))
```

Age Cohort#

	Juvenile	Young Adult	Older Adult
mean	321.6	225.2	215.0
median	154.6	109.2	90.7

```
> emmeans(mymodel, pairwise ~ cohort, adjust="holm")
```

NOTE: Results may be misleading due to involvement in interactions

$`emmeans`

cohort	emmean	SE	df	lower.CL	upper.CL
Juvenile	2.11	0.0535	3656	2.00	2.21
Young Adult	2.04	0.0478	3656	1.94	2.13
Older Adult	1.94	0.0553	3656	1.83	2.05

Results are averaged over the levels of: mon.name, sex
Confidence level used: 0.95

$contrasts

contrast	estimate	SE	df	t.ratio	p.value	
Juvenile - Young Adult	0.0675	0.0399	3656	1.692	0.0907	NS
Juvenile - Older Adult	0.1654	0.0526	3656	3.148	0.0050	**
Young Adult - Older Adult	0.0979	0.0341	3656	2.870	0.0083	**

Results are averaged over the levels of: mon.name, sex
P value adjustment: holm method for 3 tests

Table S6. Monthly mean and median **frequency of ETEs** for all collared badgers.

month	mean	median	SD	N
Jan	0.05665840	0.03225807	0.08508989	65
Feb	0.12310302	0.07142857	0.16591999	54
Mar	0.11532165	0.09677419	0.13190134	45
Apr	0.18164540	0.10000000	0.19262927	57
May	0.21504349	0.16129032	0.18854436	94
Jun	0.15814640	0.11666667	0.15124285	88
Jul	0.18333514	0.16129032	0.15581941	80
Aug	0.20013654	0.16129032	0.16778857	70
Sep	0.22873333	0.16666667	0.19514852	50
Oct	0.12543863	0.04761905	0.18497236	77
Nov	0.12265954	0.03333333	0.21075792	82
Dec	0.07054936	0.01612903	0.12639166	76
Annual mean	0.148			
Annual median		0.098		

Table S7. Monthly mean and median **ETE distance** for all collared badgers.

month	mean	median	SD	N
Jan	278.7598	207.20	283.6714	112
Feb	339.6811	186.20	398.1043	175
Mar	208.8883	135.95	233.2647	162
Apr	231.9888	125.00	346.5915	285
May	181.0494	90.40	259.5455	619
Jun	200.1591	82.40	378.8305	403
Jul	182.4752	87.80	254.1653	443
Aug	204.8509	83.85	371.5323	434
Sep	192.1787	76.00	291.9943	342
Oct	303.4925	154.80	372.9226	267
Nov	417.2844	286.90	428.2357	301
Dec	354.4782	194.40	393.4613	165
Annual mean	257.94			
Annual median		130.48		

Table S8. ANOVA results from the best (lowest AICc) model (GLMM) explaining variation in log **ETE 'investment'** (N = 3707). age cohorts used were Juvenile (age = 0, 1), Young Adult (age = 2, 3), Older Adult (age >3); month = calendar month

	**Chi Sq.**	**df**	**Pr(>Chisq)**	
(Intercept)	27.3604	1	1.69E-07	***
sex	3.5668	1	0.058946	.
age.cohort	5.7347	2	0.05685	.
month	27.5153	11	0.003839	**
sex : age.cohort	5.8031	2	0.054938	.
sex : month	49.1124	11	9.03E-07	***
age.cohort : month	171.4194	22	<2.2E-16	***
sex : age : month	139.1311	20	<2.2E-16	***

Table S9. Output from emmeans (Lenth 2018) post-hoc tests for month (as an independent term) within the model explaining variation in **ETE frequency**.

> emmeans(mymodel, pairwise ~ month, adjust="holm")
>
$`emmeans`

mon.name	emmean	SE	df	lower.CL	upper.CL
Jan	0.0526	0.0331	819	-0.0123	0.118
Feb	0.1132	0.034	819	0.0464	0.18
Mar	0.1157	0.035	819	0.047	0.184
Apr	0.2161	0.0338	819	0.1497	0.282
May	0.2322	0.0314	819	0.1705	0.294
Jun	0.1726	0.0316	819	0.1105	0.235
Jul	0.1903	0.032	819	0.1274	0.253
Aug	0.2058	0.0326	819	0.1418	0.27
Sep	0.2328	0.0342	819	0.1656	0.3
Oct	0.1365	0.0322	819	0.0734	0.2
Nov	0.13	0.032	819	0.0673	0.193
Dec	0.0782	0.0323	819	0.0148	0.142

Results are averaged over the levels of: cohort3, sex. Confidence level used: 0.95

$contrasts

contrast	estimate	SE	df	t.ratio	p.value
Jan-Feb	-0.060539	0.025	819	-2.421	0.4548
Jan-Mar	-0.063061	0.0264	819	-2.386	0.4548
Jan-Apr	-0.163443	0.025	819	-6.533	<.0001
Jan-May	-0.179591	0.0225	819	-7.975	<.0001
Jan-Jun	-0.119981	0.0228	819	-5.254	<.0001
Jan-Jul	-0.137644	0.0234	819	-5.873	<.0001
Jan-Aug	-0.153202	0.0241	819	-6.346	<.0001
Jan-Sep	-0.180155	0.0262	819	-6.871	<.0001
Jan-Oct	-0.083885	0.0235	819	-3.574	0.016
Jan-Nov	-0.077387	0.0232	819	-3.343	0.0347
Jan-Dec	-0.02562	0.0234	819	-1.094	1
Feb-Mar	-0.002522	0.0273	819	-0.092	1
Feb-Apr	-0.102904	0.026	819	-3.955	0.0039
Feb-May	-0.119052	0.0238	819	-5.008	<.0001
Feb-Jun	-0.059442	0.0241	819	-2.466	0.4282
Feb-Jul	-0.077105	0.0247	819	-3.123	0.0687
Feb-Aug	-0.092663	0.0254	819	-3.652	0.0122
Feb-Sep	-0.119616	0.0274	819	-4.37	0.0007
Feb-Oct	-0.023346	0.0248	819	-0.942	1
Feb-Nov	-0.016848	0.0246	819	-0.686	1
Feb-Dec	0.034919	0.0248	819	1.407	1
Mar-Apr	-0.100382	0.0272	819	-3.686	0.0109
Mar-May	-0.116531	0.0251	819	-4.642	0.0002

Mar-Jun	-0.05692	0.0254	819	-2.239	0.6357
Mar-Jul	-0.074583	0.0261	819	-2.86	0.1479
Mar-Aug	-0.090141	0.0267	819	-3.371	0.0322
Mar-Sep	-0.117095	0.0286	819	-4.09	0.0023
Mar-Oct	-0.020824	0.0262	819	-0.796	1
Mar-Nov	-0.014326	0.0259	819	-0.553	1
Mar-Dec	0.037441	0.0262	819	1.429	1
Apr-May	-0.016148	0.0231	819	-0.699	1
Apr-Jun	0.043462	0.0236	819	1.844	1
Apr-Jul	0.025799	0.0243	819	1.06	1
Apr-Aug	0.010241	0.025	819	0.41	1
Apr-Sep	-0.016712	0.027	819	-0.619	1
Apr-Oct	0.079558	0.0246	819	3.241	0.0472
Apr-Nov	0.086056	0.0243	819	3.54	0.0177
Apr-Dec	0.137823	0.0247	819	5.589	<.0001
May-Jun	0.059611	0.0202	819	2.958	0.1116
May-Jul	0.041947	0.021	819	2.001	1
May-Aug	0.026389	0.0218	819	1.212	1
May-Sep	-0.000564	0.0242	819	-0.023	1
May-Oct	0.095706	0.0216	819	4.429	0.0006
May-Nov	0.102205	0.0214	819	4.767	0.0001
May-Dec	0.153971	0.0219	819	7.03	<.0001
Jun-Jul	-0.017663	0.0211	819	-0.836	1
Jun-Aug	-0.033221	0.022	819	-1.512	1
Jun-Sep	-0.060175	0.0244	819	-2.467	0.4282
Jun-Oct	0.036096	0.0218	819	1.653	1
Jun-Nov	0.042594	0.0217	819	1.966	1
Jun-Dec	0.094361	0.0221	819	4.264	0.0011
Jul-Aug	-0.015558	0.0222	819	-0.702	1
Jul-Sep	-0.042511	0.0247	819	-1.722	1
Jul-Oct	0.053759	0.0222	819	2.418	0.4548
Jul-Nov	0.060257	0.0222	819	2.716	0.2228
Jul-Dec	0.112024	0.0226	819	4.948	0.0001
Aug-Sep	-0.026953	0.0252	819	-1.069	1
Aug-Oct	0.069317	0.023	819	3.018	0.0946
Aug-Nov	0.075815	0.023	819	3.3	0.0393
Aug-Dec	0.127582	0.0234	819	5.448	<.0001
Sep-Oct	0.09627	0.025	819	3.853	0.0058
Sep-Nov	0.102769	0.0249	819	4.122	0.002
Sep-Dec	0.154535	0.0253	819	6.097	<.0001
Oct-Nov	0.006498	0.0217	819	0.3	1
Oct-Dec	0.058265	0.0221	819	2.641	0.2696
Nov-Dec	0.051767	0.0215	819	2.403	0.4548

Results are averaged over the levels of: cohort3, sex.
P value adjustment: holm method for 66 tests

Table S10. Output from emmeans (Lenth 2018) post-hoc tests for month (as an independent term) within the model explaining variation in **ETE distance**.

```
> emmeans(mymodel, pairwise ~ month, adjust="holm")
>
$`emmeans`
```

mon.name	emmean	SE	df	lower.CL	upper.CL
Jan	2.46	0.0697	3672	2.32	2.6
Feb	2.26	0.0551	3672	2.15	2.37
Mar	2.17	0.0485	3672	2.08	2.27
Apr	2.09	0.0388	3672	2.01	2.17
May	2.03	0.0332	3672	1.96	2.1
Jun	2.06	0.0356	3672	1.99	2.13
Jul	2.03	0.0349	3672	1.96	2.1
Aug	2.04	0.0354	3672	1.97	2.11
Sep	1.91	0.0368	3672	1.84	1.98
Oct	2.09	0.0397	3672	2.01	2.17
Nov	2.21	0.039	3672	2.13	2.28
Dec	2.29	0.0493	3672	2.19	2.39

Results are averaged over the levels of: cohort3, sex. Confidence level used: 0.95

$contrasts

contrast	estimate	SE	df	t.ratio	p.value
Jan-Feb	0.197888	0.0775	3672	2.554	0.2779
Jan-Mar	0.286914	0.0737	3672	3.892	0.0041
Jan-Apr	0.369243	0.07	3672	5.273	<.0001
Jan-May	0.429301	0.0674	3672	6.367	<.0001
Jan-Jun	0.401885	0.0682	3672	5.892	<.0001
Jan-Jul	0.428275	0.0678	3672	6.314	<.0001
Jan-Aug	0.422822	0.0682	3672	6.197	<.0001
Jan-Sep	0.549669	0.0696	3672	7.893	<.0001
Jan-Oct	0.368879	0.0705	3672	5.231	<.0001
Jan-Nov	0.253792	0.0709	3672	3.578	0.0121
Jan-Dec	0.167745	0.0749	3672	2.241	0.6026
Feb-Mar	0.089026	0.0593	3672	1.502	1
Feb-Apr	0.171355	0.0546	3672	3.137	0.0551
Feb-May	0.231414	0.0518	3672	4.467	0.0004
Feb-Jun	0.203998	0.0531	3672	3.843	0.0047
Feb-Jul	0.230388	0.0524	3672	4.396	0.0005
Feb-Aug	0.224935	0.0532	3672	4.227	0.0011
Feb-Sep	0.351782	0.0547	3672	6.437	<.0001
Feb-Oct	0.170991	0.0558	3672	3.066	0.0656
Feb-Nov	0.055904	0.0566	3672	0.987	1
Feb-Dec	-0.030143	0.0627	3672	-0.481	1
Mar-Apr	0.082329	0.0476	3672	1.73	1
Mar-May	0.142387	0.0445	3672	3.203	0.0453

Mar-Jun	0.114971	0.0463	3672	2.483	0.3272
Mar-Jul	0.141361	0.0457	3672	3.091	0.0624
Mar-Aug	0.135908	0.0464	3672	2.929	0.0992
Mar-Sep	0.262755	0.0479	3672	5.489	<.0001
Mar-Oct	0.081965	0.05	3672	1.641	1
Mar-Nov	-0.033122	0.0503	3672	-0.659	1
Mar-Dec	-0.119169	0.0574	3672	-2.077	0.8715
Apr-May	0.060058	0.033	3672	1.823	1
Apr-Jun	0.032642	0.0367	3672	0.889	1
Apr-Jul	0.059033	0.0359	3672	1.643	1
Apr-Aug	0.05358	0.0362	3672	1.481	1
Apr-Sep	0.180426	0.0376	3672	4.798	0.0001
Apr-Oct	-0.000364	0.0411	3672	-0.009	1
Apr-Nov	-0.115451	0.0405	3672	-2.85	0.1186
Apr-Dec	-0.201498	0.0501	3672	-4.019	0.0024
May-Jun	-0.027416	0.0298	3672	-0.92	1
May-Jul	-0.001026	0.0292	3672	-0.035	1
May-Aug	-0.006479	0.0295	3672	-0.22	1
May-Sep	0.120368	0.0321	3672	3.753	0.0066
May-Oct	-0.060422	0.0363	3672	-1.666	1
May-Nov	-0.175509	0.0355	3672	-4.948	<.0001
May-Dec	-0.261557	0.0462	3672	-5.66	<.0001
Jun-Jul	0.02639	0.0313	3672	0.844	1
Jun-Aug	0.020937	0.0324	3672	0.646	1
Jun-Sep	0.147784	0.0352	3672	4.196	0.0012
Jun-Oct	-0.033006	0.0385	3672	-0.858	1
Jun-Nov	-0.148093	0.0382	3672	-3.872	0.0043
Jun-Dec	-0.234141	0.0479	3672	-4.884	0.0001
Jul-Aug	-0.005453	0.0311	3672	-0.176	1
Jul-Sep	0.121394	0.0339	3672	3.582	0.0121
Jul-Oct	-0.059396	0.0372	3672	-1.596	1
Jul-Nov	-0.174483	0.0368	3672	-4.736	0.0001
Jul-Dec	-0.260531	0.0472	3672	-5.522	<.0001
Aug-Sep	0.126847	0.034	3672	3.732	0.0069
Aug-Oct	-0.053943	0.0379	3672	-1.422	1
Aug-Nov	-0.169031	0.0372	3672	-4.55	0.0003
Aug-Dec	-0.255078	0.0476	3672	-5.355	<.0001
Sep-Oct	-0.18079	0.0383	3672	-4.721	0.0001
Sep-Nov	-0.295877	0.0383	3672	-7.721	<.0001
Sep-Dec	-0.381925	0.0494	3672	-7.735	<.0001
Oct-Nov	-0.115087	0.0397	3672	-2.896	0.1063
Oct-Dec	-0.201134	0.0499	3672	-4.034	0.0023
Nov-Dec	-0.086047	0.0475	3672	-1.812	1

Results are averaged over the levels of: cohort3, sex
P value adjustment: holm method for 66 tests

Table S11. The badger seasonal calendar, showing monthly occurrence of winter lethargy, birth of cubs, circulating testosterone, percentage of females in oestrus, long-duration matings, male ETE investment and female ETE investment. Shades of grey have been used to represent numbers, such that white = 0% and black = 100%. Sources of material are identified by superscript letters: A – this study, B– Roper 2010, C – Corner et al. 2015 and D – Neal & Cheeseman 1998.

	**Dec**	**Jan**	**Feb**	**Mar**	**Apr**	**May**	**Jun**	**Jul**	**Aug**	**Sep**	**Oct**	**Nov**
season	WINTER	WINTER	WINTER	SPRING	SPRING	SPRING	SUMMER	SUMMER	SUMMER	AUTUMN	AUTUMN	AUTUMN
winter lethargy[A]												
birth of cubs[B]												
circulating testosterone[B]												
females in oestrus[C]												
long-duration matings[D]												
male ETE investment[A]												
female ETE investment[A]												

Table S12. Monthly means of ETE frequency, ETE distance and ETE investment for Male and Female badgers across the year.

fETE	Jan	Feb	Mar	Apr	May	Jun	Jul	Aug	Sep	Oct	Nov	Dec
**Females**	0.0179	0.0437	0.0798	0.1629	0.2334	0.1413	0.1633	0.2123	0.2148	0.0978	0.0983	0.0443
**Males**	0.0920	0.1820	0.1413	0.2025	0.1959	0.1766	0.2067	0.1839	0.2451	0.1569	0.1523	0.0968

ETE dist. (m)	Jan	Feb	Mar	Apr	May	Jun	Jul	Aug	Sep	Oct	Nov	Dec
**Females**	373.8	179.7	132.5	139.8	154.1	161.7	163.3	197.4	127.9	188.2	313.0	258.8
**Males**	264.1	371.5	244.0	305.3	215.5	237.3	200.5	216.4	259.5	385.5	494.4	397.3

ETE 'invest'	Jan	Feb	Mar	Apr	May	Jun	Jul	Aug	Sep	Oct	Nov	Dec
**Females**	6.7	7.8	10.6	22.8	36.0	22.8	26.7	41.9	27.5	18.4	30.8	11.5
**Males**	24.3	67.6	34.5	61.8	42.2	41.9	41.4	39.8	63.6	60.5	75.3	38.4

Table S13. ETE summary statistics for Breeding and Non-breeding female badgers at the end of the year.

**fETE**	**Jan**	**Feb**	**Mar**	**Apr**	**May**	**Jun**	**Jul**	**Aug**	**Sep**	**Oct**	**Nov**	**Dec**
**Breeding**	0.0172	0.0479								0.1105	0.1229	0.0284
**Non-breeding**	0.0185	0.0357								0.0869	0.0768	0.0571

**ETE dist. (m)**	**Jan**	**Feb**	**Mar**	**Apr**	**May**	**Jun**	**Jul**	**Aug**	**Sep**	**Oct**	**Nov**	**Dec**
**Breeding**	304.2	182.9								164.8	360.2	444.0
**Non-breeding**	453.4	172.6								210.4	248.3	181.7

**ETE 'invest'**	**Jan**	**Feb**	**Mar**	**Apr**	**May**	**Jun**	**Jul**	**Aug**	**Sep**	**Oct**	**Nov**	**Dec**
**Breeding**	19.7	22.8								18.2	44.3	12.6
**Non-breeding**	22.9	15.0								18.3	19.1	10.4

Table S14. Plural breeding in the study area. Plural breeders are highlighted in **bold** text.

	2010	2011	2012	2013	2014	2015	2016
	Carla<br>**Dolly**<br>**Lily**	Bluebell<br>Carla<br>Debbie<br>Sheila	**Berry**<br>Debbie<br>Hazel<br>Lily<br>**Sheila**	**Berry**<br>Emma<br>Meg<br>**Sheila**	Berry<br>Cecelia<br>Cherry<br>**Ivy**<br>**Violet**	**Berry**<br>Cecilia<br>Cherry<br>Fern<br>Heather<br>**Ivy**<br>Jessie<br>Rosie<br>**Sheila**<br>**Violet**	**Berry**<br>Cherry<br>Fern<br>Ivy<br>Jessie<br>**Millie**<br>**Misty**<br>**Sheila**<br>**Snowdrop**<br>**Sylvia**
# plural social groups	1	0	1	1	1	2	3
# breeding females	3	4	5	4	5	10	10
# in same social group	2	0	2	2	2	4	6

Table S15. Saturated model to explain the variation in **ETE frequency**.

```
glmmTMB(freq.ETE ~ sex + age.cohort + sex:age.cohort + month
        + month:sex + month:age.cohort
        + (1|soc.gp/name) + (1|year),
        dispformula=~1,
        family=gaussian,
        na.action = "na.fail",
        control=glmmTMBControl(profile=quote(length(parameters$beta)>=5)),
        data=my.frequency.data)
```

Table S16. Saturated model to explain the variation in **ETE distance**.

```
glmmTMB(ete.distance ~ sex + age.cohort + sex:age.cohort + month + month:sex
        + month:age.cohort + freq.ETE + fETE:sex + fETE:month + freq.ETE:age.cohort
        + (1|name) + (1|year),
        dispformula=~1,
        family=gaussian,
        control=glmmTMBControl(optCtrl=list(iter.max=1e8,eval.max=1e8)),
        na.action = "na.fail",
        data = my.distance.data)
```
